# Overcoming the crystallization and designability issues in the ultrastable zirconium phosphonate framework system

**DOI:** 10.1038/ncomms15369

**Published:** 2017-05-30

**Authors:** Tao Zheng, Zaixing Yang, Daxiang Gui, Zhiyong Liu, Xiangxiang Wang, Xing Dai, Shengtang Liu, Linjuan Zhang, Yang Gao, Lanhua Chen, Daopeng Sheng, Yanlong Wang, Juan Diwu, Jianqiang Wang, Ruhong Zhou, Zhifang Chai, Thomas E. Albrecht-Schmitt, Shuao Wang

**Affiliations:** 1 School for Radiological and Interdisciplinary Sciences (RAD-X), Collaborative Innovation Center of Radiation Medicine of Jiangsu Higher Education Institutions, Soochow University, Jiangsu 215123, China; 2 School of Environment and Biological Engineering, Nanjing University of Science & Technology, Nanjing 210094, China; 3 Shanghai Institute of Applied Physics and Key Laboratory of Nuclear Radiation and Nuclear Energy Technology, Chinese Academy of Sciences, Shanghai 201800, China; 4 Computational Biology Center, IBM Thomas J Watson Research Center, Yorktown Heights, New York 10598, USA; 5 Department of Chemistry, Columbia University, New York, New York 10027, USA; 6 Department of Chemistry and Biochemistry, Florida State University, 95 Chieftain Way, Tallahassee, Florida 32306, USA

## Abstract

Metal-organic frameworks (MOFs) based on zirconium phosphonates exhibit superior chemical stability suitable for applications under harsh conditions. These compounds mostly exist as poorly crystallized precipitates, and precise structural information has therefore remained elusive. Furthermore, a zero-dimensional zirconium phosphonate cluster acting as secondary building unit has been lacking, leading to poor designability in this system. Herein, we overcome these challenges and obtain single crystals of three zirconium phosphonates that are suitable for structural analysis. These compounds are built by previously unknown isolated zirconium phosphonate clusters and exhibit combined high porosity and ultrastability even in fuming acids. SZ-2 possesses the largest void volume recorded in zirconium phosphonates and SZ-3 represents the most porous crystalline zirconium phosphonate and the only porous MOF material reported to survive in aqua regia. SZ-2 and SZ-3 can effectively remove uranyl ions from aqueous solutions over a wide pH range, and we have elucidated the removal mechanism.

## Abstract (web summary)

Zirconium phosphonate based metal-organic frameworks often exhibit superior chemical stabilities, but typically exist as poorly crystalline or amorphous materials. Here the authors exploit an ionothermal method to obtain highly porous and remarkably stable single crystalline zirconium phosphonate frameworks that can efficiently remove uranyl ions from aqueous solutions.

The long-term stability of metal-organic framework (MOF) compounds remains a challenge. During the past decade, significant amounts of works have been devoted to designing and synthesizing MOFs that are resistant to structural degradation induced by moisture, acids/bases, oxidants/reductants and radiation, critical features for many practical applications^1^,^2^,^3^,^4^,^5^. To achieve these goals, Pearson hard and soft acid and base theory was followed^6^,^7^. For instance, hydrolytically stable zeolitic imidazolate framework materials can be assembled by combining soft low-valence metal cations and soft N-donor ligands, with some examples that can even survive in concentrated/saturated base solutions^8^,^9^. For systems with carboxylate ligands containing hard oxo donors, hard Lewis acids, that is, metal cations with high-charge density, which are typically represented by tetravalent zirconium, have been intensively studied for the construction of stable MOFs^6^,^10^. Although debates on the hydrolytic stability of some zirconium based MOFs remain^11^,^12^, examples exhibiting decent stabilities under extremely acidic conditions (for example, concentrated HCl solutions) have been shown^13^,^14^,^15^,^16^. However, there has been lacking a single example of MOF compound that can survive all fuming acids including nitric acid, aqua regia and oleum solutions that are often required for a variety of chemical processes such as used nuclear fuel reprocessing (for example, 8 M HNO_3_).

Compared to zirconium carboxylate MOFs, tetravalent zirconium phosphonates are proposed to possess elevated stabilities owing to the higher p*K*_a_ values of the phosphonate ligands^17^. Indeed, the zirconium phosphonates reported in literatures are a class of versatile materials with promising applications such as catalyst supporters, heavy metal sorbents, as well as proton-conducting materials^18^,^19^,^20^,^21^,^22^. However, there are two key hurdles for the further development of zirconium phosphonate MOFs. First, owing to the extraordinarily strong affinity between Zr^4+^ and phosphonate ligands, the poor solubility of zirconium phosphonates often leads to rapid precipitation products with poor crystallinity or even completely amorphous solids known as unconventional MOFs, pioneered by Clearfield and Alberti^23^. Although the structures of some of these materials were partially solved by powder X-ray diffraction data, a single-crystal structure of a zirconium phosphonate MOF material with precise structural information has remained elusive. Second, most reported zirconium phosphonate MOFs are based on inorganic zirconium phosphate 1D chains or 2D sheets and there has been lacking a zero-dimensional zirconium phosphate cluster that can act as the secondary building unit (SBU), leading to poor designability of the zirconium phosphonate MOF system^24^. By contrast, Zr_6_ clusters are found in several benchmark zirconium carboxylate MOFs UiO-66, PCN-222, MOF-525 and other compounds^13^,^25^,^26^,^27^,^28^,^29^, while NU-1000 contains a Zr_8_ cluster as the SBU^30^. As another consequence, zirconium phosphonates with 3D framework structures are quite rare^24^,^31^,^32^,^33^. Among these, only UPG-1, whose structure was solved by powder X-ray diffraction recently, shows permanent porosity^33^.

To overcome these issues, we pursued two strategies in our synthetic efforts: first, a sterically demanding spacer molecule was adopted to prevent the formation of a 2D structure stemming from the stacking of the layers that was recently reported by Taddei *et al*. in Zrbtbp constructed using a triangular planar ligand, 1,3,5-tris(4-phosphonophenyl)benzene (H_6_btbp)^34^. Alternatively, tetrakis[4-(dihyroxyphosphoryl)phenyl]methane (TppmH_8_) and 1,3,5,7-tetrakis(4-phosphonophenyl)adamantine (TppaH_8_) with coordination-available phosphonate groups located at four tetrahedral vertexes as the linker was selected to more effectively prevent the formation of the undesired, densely packed structures. Second, ionothermal synthesis was used to avoid the issue of hydrolysis/solvation of Zr^4+^ in the traditional hydrothermal/solvothermal reactions and to slow down the crystallization kinetics and thereby promote the growth of large single crystals. Indeed, the ionothermal reaction of ZrCl_4_ with TppmH_8_ or TppaH_8_ in the ionic liquid N-butyl-N-methylpyrrolidinium bromide ([C_4_mpyr][Br]) and N-ethylpyridinium bromide ([C_2_py][Br]) in the presence of HF afforded three highly crystalline three-dimensional (3D) microporous Zirconium(IV) phosphonates with crystal sizes that are sufficiently large for X-ray analysis. All these compounds are built based on zero-dimensional zirconium phosphate cluster as the SBU, initially opening the way for the reticular design and synthesis in the zirconium phosphonate system. In addition, these compounds exhibit ultrastabilities in acid solutions especially in fuming acids. Furthermore, two of these compounds possess high permanent porosities and excellent uranium uptake capabilities, while one compound is the most porous crystalline zirconium phosphonate reported up to date and is the only porous MOF that survives in aqua regia.

## Results

### Structure and characterizations of SZ-1

**Figure 1:**
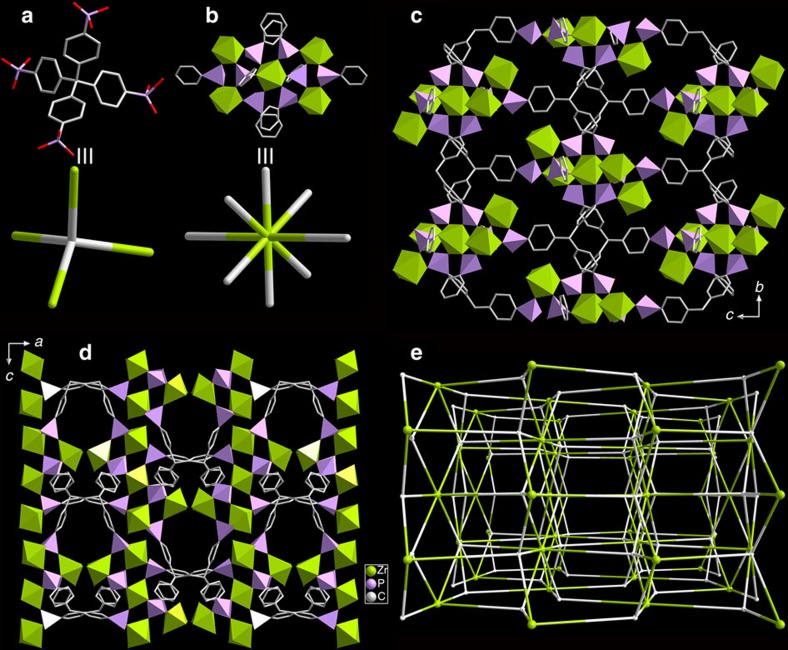
Crystal structure and network topology of SZ-1. The Tppm ligand (**a**) is connected to four 8-connected Zr_5_P_8_ clusters (**b**) to generate a 3D network with a sieve-like layered structure in the *bc* plane (**c**–**e**). Colour scheme: Zr, green; P, purple; C, grey.

[C_4_mpyr][Zr_2.5_(TppmH_3_)F_6_]·2.5H_2_O (Soochow University Zirconium Phosphonate No. 1, SZ-1) crystallizes in a centrosymmetric orthorhombic space group *Pbca*. Zr^4+^ ions are located in two coordination spheres of ZrO_6_ and ZrO_3_F_3_, which are surrounded by six and three bridging CPO_3_ groups from the Tppm ligands, respectively, as guided by strategy 1. ZrO_6_ is bridged to four ZrO_3_F_3_ polyhedra through six phosphonate groups via corner-sharing (Supplementary Tables 1 and 2). Two pairs of ZrO_3_F_3_ are further bridged by one CPO_3_ group each to give a previously unknown eight-connected Zr_5_P_8_ cluster (Fig. 1a,b, Supplementary Fig. 11 and Supplementary Table 5) containing no oxide/hydroxide, consistent with the goal of strategy 2. The Zr_5_P_8_ clusters are finally linked by the organic moieties of the Tppm ligands, resulting in a 3D anionic framework structure with [C_4_mpyr]^+^ cations filled in the pores (Fig. 1c,d). The position of [C_4_mpyr]^+^ can be perfectly determined, suggesting a strong interaction between [C_4_mpyr]^+^ and the anionic framework. There are two types of interconnected channels in the SZ-1 structure. The first is a continuous channel along the [010] direction, with window dimensions of ∼5.4 × 5.8 Å, and the second, smaller aperture is along the [100] direction. The total void volume of SZ-1 estimated using PLATON is 27.7%, which is smaller than that of the first microporous compound UPG-1 with a void volume of 32.8% (ref. 33). The SBU of Zr_5_P_8_ in SZ-1 is substantially different from the inorganic chains constructed by ZrO_6_ and phosphonate groups in UPG-1, resulting in a unique {4^10^.6^14^.8^4^}{4^5^.6}2 alb topology containing a Zr_5_P_8_ cluster as an eight-connected node and the Tppm ligand as a four-connected node (Fig. 1e).

The porosities of SZ-1 were investigated by sorption isotherms of N_2_ and CO_2_ (Supplementary Fig. 16). The results show that SZ-1 exhibits moderate porosity with a N_2_ Brunauer–Emmett–Teller (BET) and Langmuir surface area of 10.2 and 16.5 m^2^ g^−1^, respectively, likely because of the difficulty of exchanging [C_4_mpyr]^+^. The fuming acid solutions with large excess of protons were used to exchange the [C_4_mpyr]^+^, but this was unsuccessful as the surface area did not change significantly. Nevertheless, this observation provides a direct evidence for the ultrastability in fuming acid as discussed below.

**Figure 2:**
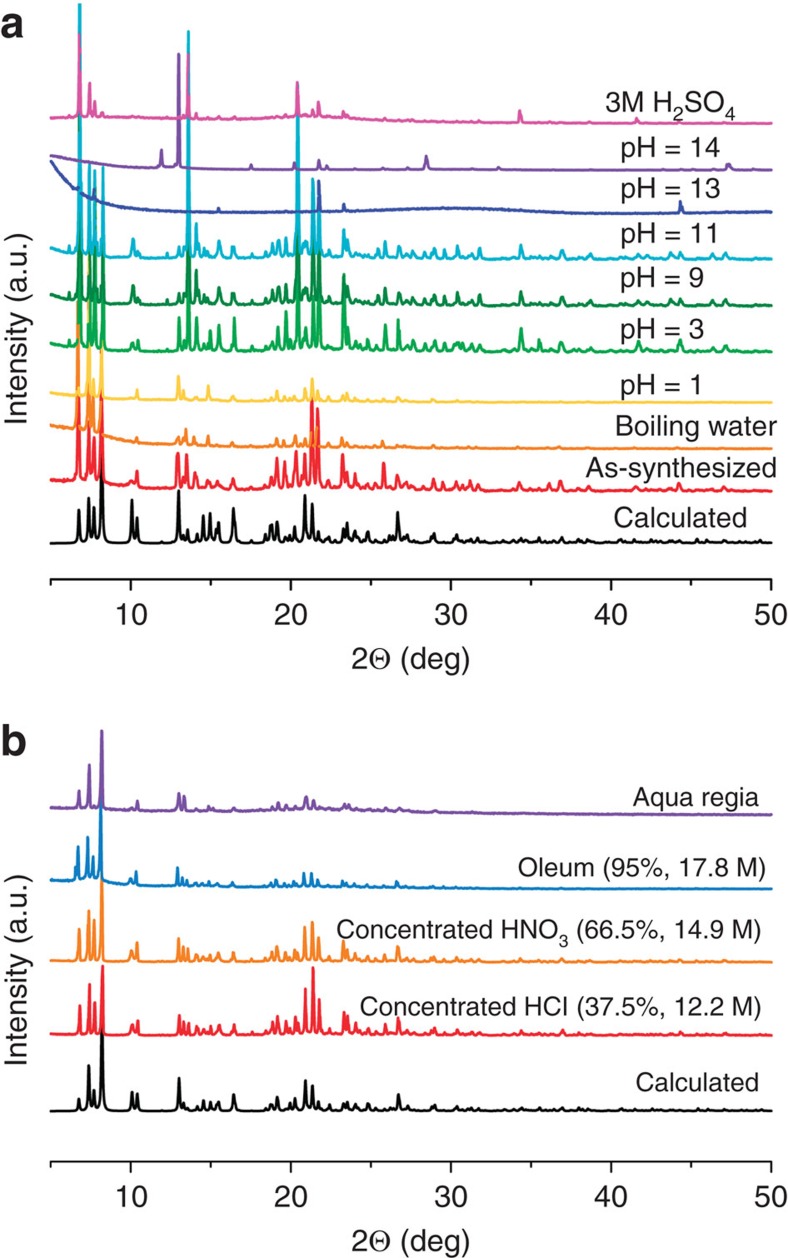
Powder X-ray diffraction of SZ-1. Powder X-ray diffraction spectra for compound SZ-1 after soaking in various solutions (**a**) and in fuming acids (**b**).

The thermal behaviour of SZ-1 was evaluated by thermogravimetric analysis under nitrogen flow, revealing that SZ-1 is stable up to 300 °C, as further demonstrated by temperature-dependent X-ray diffraction measurements (Supplementary Figs 17 and 18). As one of the most important features, the hydrolytic stabilities of SZ-1 were tested in aqueous solutions. As expected, the crystallinity of the compounds was completely preserved in boiling water and aqueous solutions over a wide pH range (1–11) (Fig. 2a). More impressively, SZ-1 can survive in a variety of fuming acids including 12.2 M HCl, 14.9 M HNO_3_, aqua regia and oleum (that is, 17.8 M H_2_SO_4_) solutions (Fig. 2b). Furthermore, this decent stability in fuming acids was further checked by solid sample mass maintain test confirming that SZ-1 is completely insoluble in these solutions. Furthermore, the crystal structures can be well refined using the diffraction data collected on the oleum/aqua regia-soaked SZ-1 crystals, which are identical with the original structure (Supplementary Fig. 20 and Table 6). This finding is significant since no MOF material has yet been reported to survive in all these fuming acids^16^. By contrast, BUT-12, 13 and PCN-222 have been shown to survive in concentrated HCl solutions^13^,^14^; MOF-808 maintains its crystallinity in 0.1 M H_2_SO_4_ solution^27^; MOF-525 and MOF-545 are stable in water/acetic acid (50/50, vol) solution for one day^26^; PCN-225 and PCN-230 show excellent stabilities in aqueous solutions over a wide pH range (0–12)^3^,^15^; while UPG-1 was shown to be stable under 0.1 M HCl solution at 120 °C for three days^33^.

### Structure and characterizations of SZ-2

**Figure 3:**
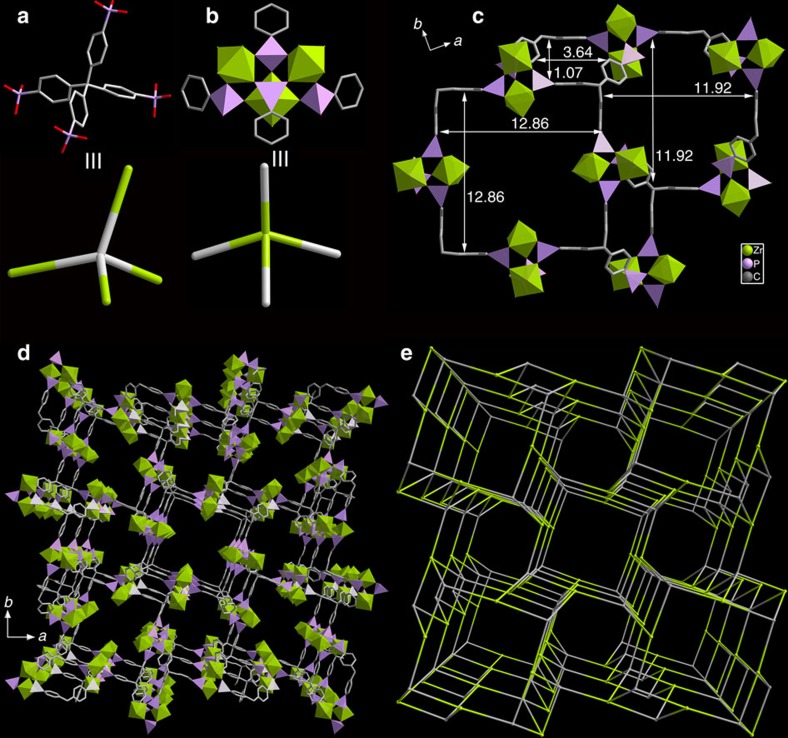
Crystal structure and network topology of SZ-2. The Tppm ligand (**a**) is connected to four 4-connected Zr_3_P_4_ clusters (**b**) to generate a 3D network with three types of windows of different sizes along the [001] direction (**c**–**e**).

Although the stability of SZ-1 is quite promising, the porosity is still limited, which needs enhancement for further applications. By using more amount of ionic liquids aiming at the expansion of pores, another microporous negative charged 3D framework compound, [C_4_mpyr]_2_[Zr_1.5_(Tppm)_0.5_F_4_·6H_2_O] (SZ-2), was obtained as large single crystals (Supplementary Fig. 13). The synthesis of pure SZ-2 material is found to be very challenging as it often co-crystallizes with SZ-1 under a variety of reaction conditions. To resolve this issue, we introduced a unique technique of slow diffusion into the ionothermal reaction to further slow down the crystallization kinetics (as shown in Supplementary Fig. 10), which allowed us to successfully obtain the pure SZ-2 product as confirmed by the powder X-ray diffraction data. SZ-2 crystallizes in a centrosymmetric tetragonal space group *I*4/*m* and is much more porous than SZ-1, as calculated by PLATON. ZrO_3_F_3_ and ZrO_4_F_2_ moieties are found in SZ-2 and are coordinated by three and four Tppm ligands, respectively (Fig. 3a, Supplementary Tables 1 and 3). Two ZrO_3_F_3_ and one ZrO_4_F_2_ polyhedra are bridged by four bridging CPO_3_ groups, resulting in a unique four-connected Zr_3_P_4_ cluster (Fig. 3b), and a 3D microporous framework structure is achieved through the linkage of the Tppm ligands with highly disordered C_4_mpyr^+^ cations filled in the 3D channels (Fig. 3c,d). Notably, the special arrangement of coordination of Zr_3_P_4_ cluster with the organic moiety results in a significant distortion of the central carbon atom coordination in the Tppm ligand deviating from the ideal tetrahedral configuration. Further inspection of the structure reveals that this material should be a good candidate for ion-exchange applications. Three types of apertures are found along the [001] direction, with sizes of 12.86 × 12.86, 11.92 × 11.92 Å, and 3.64 × 1.07 Å (after subtraction of vdW radii of C atoms). The window size for channels along the [100] and [010] directions are close to 1 nm in diameter. The total void volume of SZ-2 is 68.2%, which is the largest reported for a zirconium phosphonate MOFs and is significantly larger than those observed previously. Topological analysis shows that Zr_3_P_4_ cluster serves as a four-connected node and the Tppm ligand as a 4-connected node, further leading to a 4-c net with atn topology and a topological point symbol of {4^2^.6^3^.8} (Fig. 3e).

**Figure 4:**
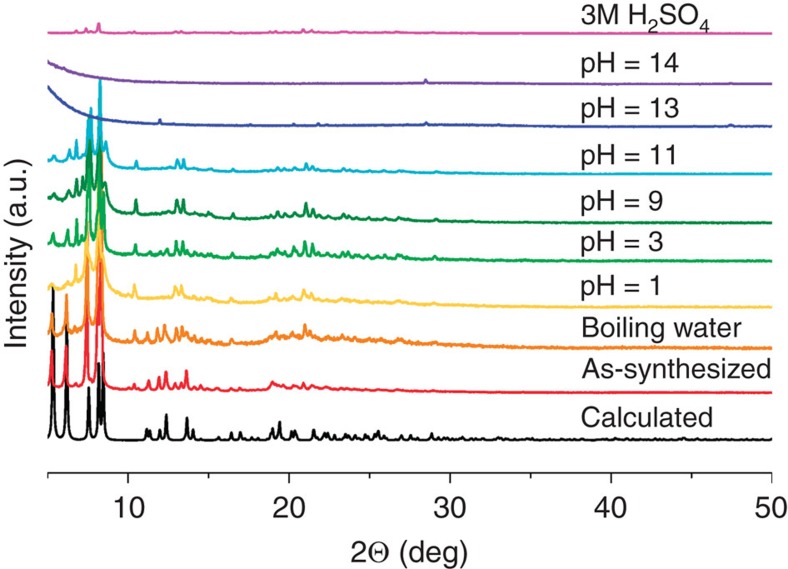
Powder X-ray diffraction of SZ-2. Powder X-ray diffraction spectra for compound SZ-2 after soaking in various solutions.

N_2_ adsorption of SZ-2 shows a significantly elevated surface area of 225 (BET) and 375 (Langmuir) m^2^ g^−1^, respectively (Supplementary Fig. 16), compared to that of SZ-1. These values are still lower than those calculated based on the void volume of SZ-2, likely due to the charge-balancing cations in the pore and/or partial framework collapse during the measurement. In comparison, the Langmuir and BET surface area of the neutral framework UPG-1 is 410 and 514 m^2^ g^−1^, respectively, as measured using both carbon dioxide and n-butane, while the N_2_ adsorption measurement did not yield a noticeable uptake value^33^. The thermal behaviour of SZ-2 was also measured, showing that SZ-2 is stable up to ca. 300 °C (Supplementary Fig. 17). The hydrolytic stabilities tests of SZ-2 reveal that the crystallinity of SZ-2 was completely preserved in boiling water and aqueous solutions over a wide pH range (1–11) (Fig. 4). However, SZ-2 is not able to survive fuming acids, which may be a consequence of the highly distorted central carbon atom coordination in the Tppm ligand.

### Structure and characterizations of SZ-3

**Figure 5:**
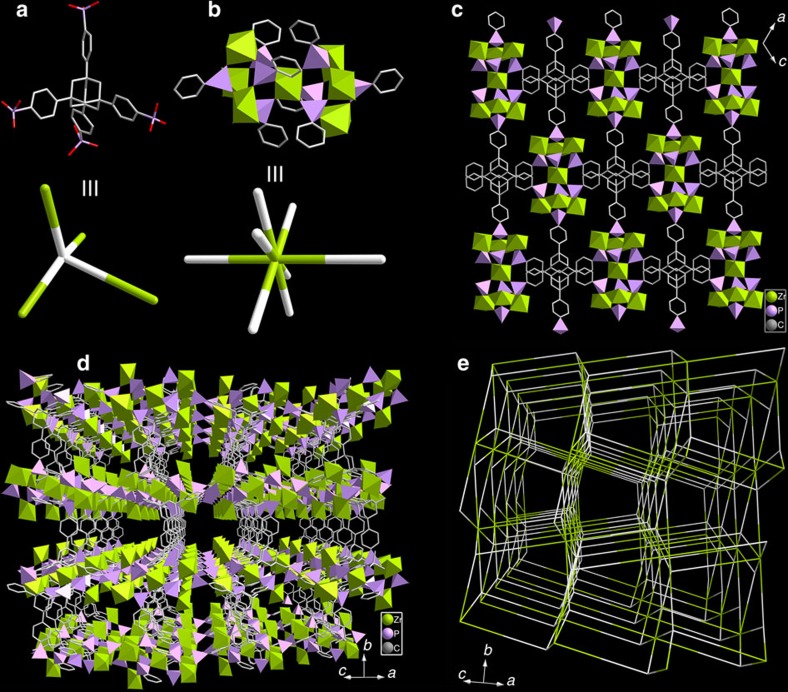
Crystal structure and network topology of SZ-3. The Tppa ligand (**a**) is connected to four 4-connected Zr_7_P_8_ clusters (**b**) to generate a 3D network with hybrid porous layers in *ac* plane (**c**), which are linked by the organic moieties along *b* axis (**d**,**e**).

Although SZ-1 and SZ-2 exhibit advantages in terms of ultrastability in fuming acids and high porosity respectively, these features do not yet combined in one compound. To solve this issue, we switched ligand from Tppm to Tppa with the change of ligand central portion from a single carbon atom to a much larger adamantane, aiming at reducing the effect of ligand distortion. Indeed, the key compound [C_2_py]_2_[Zr_3.5_(TppaH)F_9_]·6.5H_2_O (SZ-3) that is featured with both ultrastability in fuming acids and high porosity was successfully obtained. SZ-3 crystallizes in a monoclinic space group *P*2(1)*/n* (Supplementary Tables 1 and 4). Zr^4+^ ions in SZ-3 are also located in two coordination spheres of ZrO_6_ and ZrO_3_F_3_, which are surrounded by six and three bridging CPO_3_ groups from the Tppa ligands, respectively (Fig. 5a). Six ZrO_3_F_3_ polyhedra are connected to one ZrO_6_ through six corner-sharing phosphonate groups, giving a sandglass shaped structure terminated by a phosphonate bridging three ZrO_3_F_3_ polyhedra. This further gives rise to another previously unknown eight-connected Zr_7_P_8_ cluster (Fig. 5b). The Zr_7_P_8_ clusters are linked by the organic moieties of the Tppa ligands, resulting in a new anionic 3D framework structure with disordered [C_2_py]^+^ cations filled in the pores (Fig. 5c,d). The total void volume of SZ-3 is 46.5%, which is the second largest among all crystalline zirconium phosphonates with known crystal structures, only smaller than SZ-2 in this work. Although the SBU of Zr_7_P_8_ in SZ-3 is substantially different from that in SZ-1, the {4^10^.6^14^.8^4^}{4^5^.6}2 alb-4,8-Pbcn topology is the same with that of SZ-1, containing a Zr_7_P_8_ cluster as an eight-connected node and the Tppa ligand as a four-connected node (Fig. 5e).

**Figure 6:**
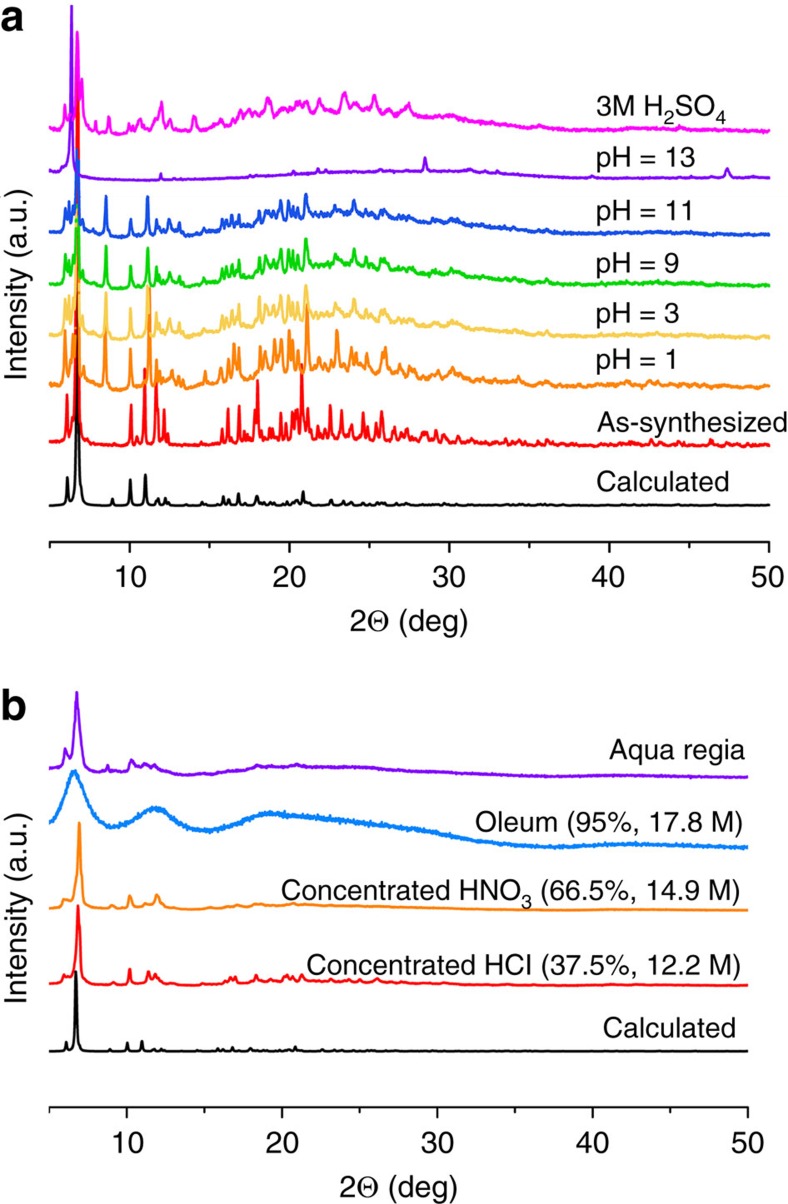
Powder X-ray diffraction of SZ-3. Powder X-ray diffraction spectra for compound SZ-3 after soaking in various solutions (**a**) and in fuming acids (**b**).

The surface area of SZ-3 is evaluated by N_2_ adsorption, showing a significantly elevated surface area of 572 (BET) and 695 (Langmuir) m^2^ g^−1^, respectively (Supplementary Fig. 16), which is noticeably larger than all other crystalline zirconium phosphonate compounds reported up to date. The thermal behaviour of SZ-3 was also measured, showing that SZ-3 is stable up to ca. 210 °C (Supplementary Fig. 17). The hydrolytic stabilities tests of SZ-3 reveal that the crystallinity of SZ-3 was completely preserved not only in aqueous solutions from pH 0 to 11 (Fig. 6a), but also in fuming acids including aqua regia, concentrated HCl, and concentrated HNO_3_, making SZ-3 currently the only porous MOF material that is reproted to be able to survive aqua regia (Fig. 6b). The N_2_ adsorption of SZ-3 sample soaked in aqua regia was also tested, giving BET and Langmuir surface area of 594 and 732 m^2^ g^−1^, respectively, which are even slightly higher than those of the original sample (Supplementary Fig. 19).

### Uranyl uptake experiments and mechanism elucidation

**Figure 7:**
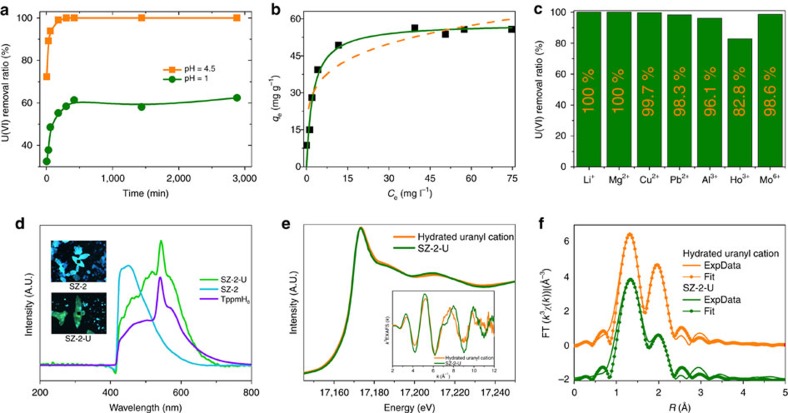
Uranyl sorption experiment results using SZ-2. (**a**) Effect of contact times on uranyl(VI) sorption onto SZ-2 at pH 4.5 and 1.0 under stirring, with C_0_=10 p.p.m., mV^−1^=1 mg ml^−1^; (**b**) the sorption data fitted by Langmuir (green solid) and Freundlich (yellow dash) models at pH=4.5, respectively; (**c**) competitive sorption of coexistent ions on SZ-2 at pH=4.5, with molar ratio of metal ions to uranyl cations are ∼10 times; (**d**) fluorescence spectra of SZ-2 (blue) and SZ-2-U (yellow-green) excited by 365-nm light before and after uranyl adsorption; (**e**) XANES and EXAFS (inset) spectra of SZ-2-U, compared with the hydrated uranyl cation in aqueous solutions; (**f**) Fourier-transformed space (*R* space) spectra of SZ-2-U, compared with the hydrated uranyl cation in aqueous solution.

Inorganic zirconium phosphates and other materials have been intensively investigated as ion-exchange materials for the removal of heavy metal contamination, especially uranium and the fission product caesium, for spent nuclear fuel partitioning and contamination remediation^21^,^35^,^36^,^37^,^38^,^39^,^40^,^41^,^42^. With their significantly improved porosity and surface area, all these three zirconium phosphonate MOFs could be promising candidates for these applications. The uranyl(VI) ion uptake results at various pH show that SZ-2 is able to almost completely remove the uranyl ions over a wide pH range from 3 to 7, while SZ-1 does not exhibit a noticeable ion-exchange capability (Supplementary Fig.21). Common ion-exchange materials, such as inorganic zirconium phosphates often lose their uranium-uptake ability below pH 2 (refs 34, 35, 36, 37, 38, 39, 40, 41). Remarkably, the uranium removal percentage can reach 62.4% using SZ-2 at pH 1.0 (Fig. 7a). This rarely observed result is likely attributed to the combined advantages of both enhanced stability and elevated porosity. The sorption kinetics of uranyl(VI) ion with SZ-2 was also evaluated. At pH 4.5, 72.3% of uranyl ions was removed within only 5 min and the sorption equilibrium with ca. 100% removal ratio was achieved within 3 h (Supplementary Fig. 22). At pH 1.0, the sorption equilibrium can be reached within 5 h, which is typical for ion-exchange materials. Sorption isotherm data fitted by the Langmuir model gives the saturated uranium sorption capacity of 58.18 mg g^−1^ for SZ-2, which is quite promising compared to the zirconium phosphate based sorbent materials (Fig. 7b). The uranyl sorption selectivity by SZ-2 was also checked to be excellent, as 10 p.p.m. of uranyl ion can be still almost completely removed, when 10 times molar excess of common competing cations are present except for the case of Ho^3+^, where the removal percentage was reduced to 82.8% (Fig. 7c, Supplementary Tables 7 and 8). This is due to the similar effective charge density between UO_2_^2+^ and Ho^3+^. In addition, intensive greenish emission can be probed for the uranium-sorbed single crystals of SZ-2 when excited by 365 nm light, revealing a sharp difference from the blue emission of SZ-2 without uranium (Fig. 7d). To investigate the uranium uptake mechanism, integrated analysis of X-ray absorption near edge structure (XANES) and extended X-ray absorption fine structure (EXAFS) on a uranium-sorbed sample of SZ-2 were measured, giving similar spectra with that obtained from aqueous solutions containing hydrated uranyl(VI) cation (Fig. 7e,f; Supplementary Table 9). Therefore, the ion-exchange process between C_4_mpyr^+^ and hydrated uranyl cations may play a major role in the sorption mechanism, which is also confirmed by X-ray photoemission spectroscopy (XPS) (Supplementary Table 10) analysis suggesting the presence of a single uranyl(VI) hydrate component in the uranyl-sorbed sample (Supplementary Fig. 23).

**Figure 8:**
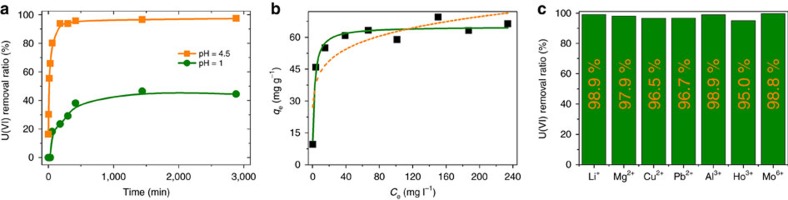
Uranyl sorption experiment results using SZ-3. (**a**) Effect of contact times on uranyl(VI) sorption onto SZ-3 at pH 4.5 and 1.0 under stirring, with C_0_=10 p.p.m., mV^−1^=1 mg ml^−1^; (**b**) the sorption data fitted by Langmuir (green solid) and Freundlich (yellow dash) models at pH=4.5, respectively; (**c**) competitive sorption of coexistent ions on SZ-3 at pH=4.5, with molar ratio of metal ions to uranyl cations are ∼10 times.

SZ-3 was also evaluated for uranium uptake showing a similar capability with that of SZ-2. As shown in Figure 8, the uranium removal percentage can reach 46.4% and 97.4% at pH 1.0 and 4.5, respectively. The sorption kinetics of uranyl(VI) ion with SZ-3 was also evaluated. At pH 4.5, 80.2% of uranyl ions was removed within 60 min and the sorption equilibrium with ca. 97.4% removal ratio was achieved within 3 h (Fig. 8a). At pH 1.0, the sorption equilibrium can be reached within 7 h. Sorption isotherm data fitted by the Langmuir model gives the saturated uranium sorption capacity of 58.18 mg g^−1^ for SZ-3 (Fig. 8b). The uranyl sorption selectivity by SZ-3 is also similar to that of SZ-2, as 10 p.p.m. of uranyl ion can be still almost completely (>95%) removed when 10 times molar excess of common competing cations are present (Fig. 8c).

### Molecular dynamics simulations on uranyl uptake

**Figure 9:**
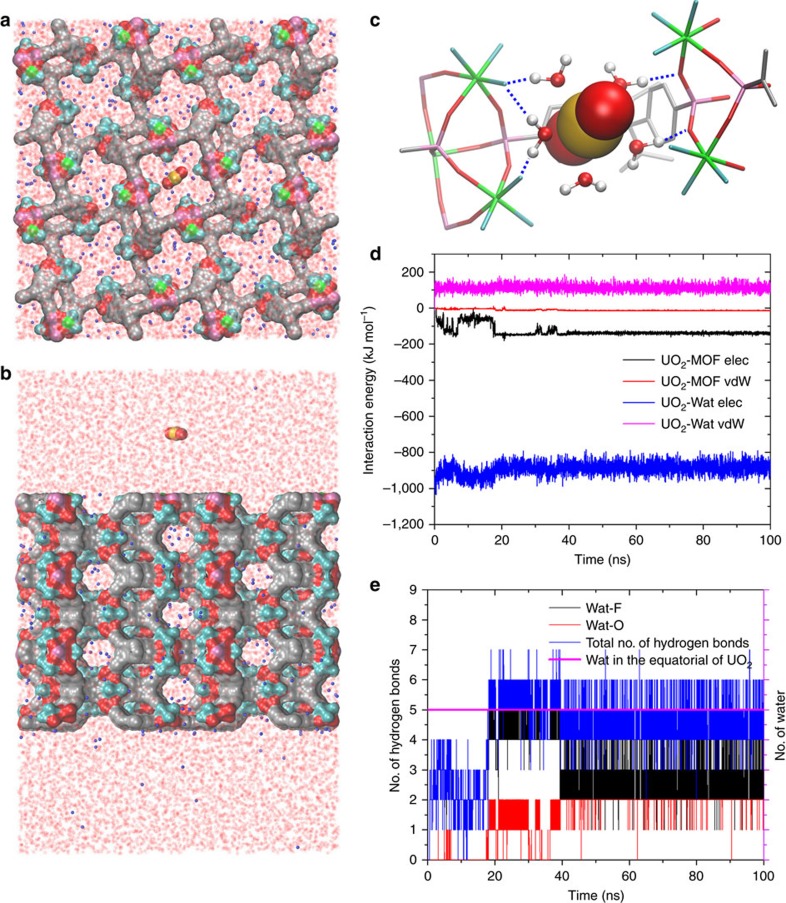
MD simulations on the process of uranyl sorption into SZ-2. The top (**a**) and side (**b**) view of the simulation system-1 (uranyl cation approaching along the *c* axis); (**c**) the final snapshot (at *t*=100 ns) of run 1 (out of total 6) to show the importance of equatorial water of uranyl cation in mediating its binding to the SZ-2 (the blue dash line denotes the hydrogen bond between equatorial water molecules and the dangling hydrogen bond acceptors); (**d**) time evolution of the electrostatic and vdW interaction energies of uranyl cation with SZ-2 and water; (**e**) the number of equatorial water molecules of uranyl cation (pink curve) and the number of hydrogen bonds formed between equatorial coordinating water molecules and other acceptors (including F and O in main framework) as the function of simulation time.

Moreover, all-atom molecular dynamics (MD) simulations were further performed to investigate the adsorption process of uranyl cation into SZ-2 and its underlying molecular mechanism. To enhance sampling, two orthogonal initial configurations were adopted to mimic the two different directions of uranyl cation adsorption: one from the top ([001] direction, Fig. 9a,b) and one from the side ([100] direction, Supplementary Fig. 26c,d). Each configuration system was simulated for six independent runs, with each 100 ns, resulting in a total aggregate simulation time of 1.2 μs. These extensive simulations reveal that the equatorial water molecules in the hydrated uranyl cation played a crucial role in mediating its binding with SZ-2 through a hydrogen bond network with those dangling hydrogen bonding acceptors from the main framework in SZ-2, such as F and O atoms that originally bind to Zr and P (Fig. 9c, Supplementary Figs 27, and 28).

To further identify the driving forces for this fast sorption kinetics, we computed time evolutions of nonbonded interactions energies (including both vdW and electrostatic) between uranyl cation with SZ-2 and water (Fig. 9d). At the first 5 ns, uranyl cation was rapidly sorbed into the framework of SZ-2. During this period, the electrostatic interaction energy between uranyl cation and SZ-2 decreased from 0 to ∼−150 kJ mol^−1^ (Fig. 9d, black curve), and the vdW interaction energy lowered from 0 to ∼−5 kJ mol^−1^ (Fig. 9d, red curve), respectively. Meanwhile, the electrostatic interaction energy between uranyl cation and water increased from −1000 to∼−900 kJ mol^−1^, and the vdW interaction energy nearly maintained at a constant value at ∼108 kJ mol^−1^. This indicates that the favourable direct nonbonded interactions between uranyl cation and SZ-2 (totally gains ∼155 kJ mol^−1^ energy) can overcompensate for the penalty in the unfavourable interactions between uranyl cation and water (totally loses ∼100 kJ mol^−1^ energy), when an uranyl cation was captured by SZ-2. After entering the pores of SZ-2, few hydrogen bonds (2.38±1.03 hydrogen bonds on average, Fig. 9e blue curve) quickly formed by the equatorial water molecules in the hydrated uranyl cation with the hydrogen bonding acceptors from the main framework in SZ-2. This is thermodynamically unfavourable and a round of configuration re-adjustment (from ∼5 ns to 17 ns) quickly occurred driven by formation of a much denser hydrogen bond network through uranyl cation's equatorial water molecules with the dangling hydrogen bonding acceptors in SZ-2. During this process, the electrostatic interaction energy between uranyl cation and SZ-2 even undergoes a remarkable increase (increased to∼−50 kJ mol^−1^, Fig. 9d black curve). Nevertheless, after ∼17 ns, along with the formation of more hydrogen bonds (4.15±0.79 hydrogen bonds on average, Fig. 9e blue curve) between equatorial water molecules and the dangling hydrogen bonding acceptors in SZ-2, the electrostatic interaction energy between uranyl cation and SZ-2 again decreased to ∼−150 kJ mol^−1^ (Fig. 9d black curve). Therefore, the strong electrostatic interaction between uranyl cation and SZ-2 effectively drives uranyl cations into the framework of SZ-2, while formation of a much denser hydrogen bond network results in a remarkably tight trapping of uranyl cation. These observations obtained from the MD simulations provided a microscopic picture of the binding with atomic details, which also support our infrared spectra analyses of SZ-2 and SZ-2-U (Supplementary Fig. 24) and the proposed ion-exchange mechanism.

## Discussion

In conclusion, by utilizing a tetra-phosphonate ligand with a sterically demanding spacer and ionothermal synthesis to modify the crystallization kinetics, we obtained three highly crystalline, microporous and ultrastable zirconium phosphonate MOF compounds, the structures of which can be facilely solved by single crystal X-ray diffraction. Notably, all three compounds are built based on zero-dimensional clusters acting as the node, resembling typical SBUs found in carboxylate-based MOFs^43^,^44^, potentially making the concept of reticular chemistry available in the zirconium phosphonate system^45^. This is also responsible for the enhanced structural porosities observed in all three compounds compared to the previously reported zirconium phosphonate MOFs. SZ-1 represents one of the most stable MOF materials in fuming acids reported to date, and SZ-2 exhibits a superior potential for the removal of uranyl(VI) ions from aqueous solutions over a wide pH range, representing a clear improvement over the traditional inorganic zirconium phosphate ion-exchange materials, especially in the low pH region. The combined high porosity and ultrastability in fuming acids is achieved in SZ-3 with the aid of ligand distortion reduction. We believe this work may open up a new avenue to create a series of highly robust MOFs materials based on high-valence metal cations and phosphonate ligands that are currently facing a significant crystallization and designability issue but may find various potential applications under harsh conditions.

## Methods

### Materials and measurements

All chemicals were obtained from commercial sources (J&K Chemical, Sinopharm Chemical Reagent, Alfa Aesar, and Adamas Company) and used without further purification. ^1^H-NMR spectra were measured using a Unity INOVA 400 instrument. Single crystal X-ray diffraction data were collected on a Bruker D8-Venture single crystal X-ray diffractometer equipped with a Turbo X-ray Source (Mo–Kα radiation, λ=0.71073 Å) adopting the direct-drive rotating anode technique. Powder X-ray diffraction patterns were measured on a Bruker D8 advance X-ray diffractometer with Cu–Kα radiation (λ=1.54056 Å) equipped with a Lynxeye one-dimensional detector. X-ray absorption spectra were collected at the beamline 14 W of the Shanghai Synchrotron Radiation Facility. Scanning electron microscopy (SEM) images and energy-dispersive spectroscopy (EDS) data were collected on a FEI Quanta 200FEG instrument. Fluorescence spectra were obtained on a Craic Technologies microspectrophotometer. Infrared spectra were collected on a Thermo Scientific Nicolet iS50 FT-IR instrument at room temperature. The concentration of uranium was determined using a Thermo Finnigan high resolution magnetic sector Element 2 inductively coupled plasma mass spectrometer (ICP-MS). The XPS data were recorded on a PHI Quantera SXM spectrometer using monochromatic Al K_α_ (1486.6 eV) X-ray radiation at room temperature. The anode was operated at 24.2 W with a typical spot size of 100 μm. The U 4 f data were analysed with MultiPak software using the iterated Shirley background and the asymmetric peak profile for both primary and satellite peaks.

Colourless crystals of three compounds were mounted on Cryoloops with paratone and optically aligned by a digital camera on a Bruker D8-Venture single crystal X-ray diffractometer. The diffraction data were collected by a CMOS area detector with a Turbo X-ray Source adopting the direct-drive rotating anode technique. The data was reduced by SAINTplus package of Bruker and the structures were solved by the direct method and refined on F^2^ by full-matrix least-squares methods using SHELXTL (2016)^46^. All the non-hydrogen atoms were refined anisotropically and hydrogen atoms except those attached to water molecules, cations of ionic liquid or disordered carbons were located on calculated positions. The positional disorder is found in Tppm-ester and SZ-1 compounds, which results in relatively large values of R_1_ and wR_2_, some C level alerts in the checkcif reports, and a B level alert in compound Tppm-ester. For SZ-2, cations of ionic liquid and solvent molecules in the channels are highly disordered and impossible to refine using conventional discrete-atom models. Therefore, contributions of these molecules were removed using SQUEEZE routine of PLATON^47^; Structure of SZ-2 was then refined again using the data generated. For SZ-3, phenomenon of pseudo-merohedral twins was found, and was solved in monoclinic space group and contributions of disordered ionic liquid molecules were removed using SQUEEZE routine of PLATON^47^. CHN elemental and thermogravimetric analysis were used to determine the formula of those compounds.

### Synthesis of TppmH_8_ and TppaH_8_

The synthesis route of TppmH_8_ and TppaH_8_ are shown in Supplementary Figs 1 and 2. The ^1^HNMR and infrared spectra for the synthesis are listed in Supplementary Figs 3, 4, 5, 6, 7, 8 and 9.

### Synthesis of SZ-1

A mixture of ZrCl_4_ (0.0625, mmol, 0.0172, g), TppmH_8_ (0.025 mmol, 0.0162, g), N-butyl-N-methylpyrrolidinium bromide [C_4_mpyr][Br] (0.2 mmol, 0.0442, g) and 5 drops of HF (40%), was placed in a 15 ml Teflon–lined stainless steel vessel and heated at 180 °C for 3 days and then cooled to 25 °C. Colourless plate crystals were collected as a pure phase (Supplementary Figs 12 and 14). Yield: 51.4% (based on zirconium). Elemental analysis for SZ-1 (C_34_H_36_F_6_NO_12_P_4_Zr_2.5_) (Calc.: C: 36.57, H: 3.25, N: 1.25; Found: C: 37.09, H: 4.049, N: 1.516.)

### Synthesis of SZ-2

SZ-2 was synthesized by the diffusion method shown in Supplementary Fig. 10: ZrCl_4_ and TppmH_8_ ligand were initially placed at the opposite sides of the reaction vessel without contact while the solid of ionic liquid [C_4_mpyr][Br] (2 mmol, 0.4421, g) and HF (2 drops) were located in the middle. As the temperature is evalated, ionic liquid is liquified and dissolves ZrCl_4_ and TppmH_8_ ligand, which slowly diffuse into each other and react. This substantially avoids the fast crystallization of SZ-1 products and results in the formation of SZ-2, as a pure phase with the yield of about 32.7% (based on zirconium, Supplementary Figs 13 and 15).

### Synthesis of SZ-3

A mixture of ZrCl_4_ (0.0625, mmol, 0.0174, g), TppaH_8_ (0.025 mmol, 0.0193, g), N-ethylpyridinium bromide [C_2_py][Br] (2.1 mmol, 0.3950, g), and 2 drops of HF (40%), was placed in a 15 ml Teflon-lined stainless steel vessel and heated at 160 °C for 3 days and then cooled to 25 °C. Colourless block crystals were collected as a pure phase. Yield: 43.6% (based on zirconium). SZ-3 (C_48_H_61_F_9_N_2_O_18.5_P_4_Zr_3.5_) (Calc.: C: 36.58, H: 3.90, N: 1.78; Found: C: 36.34, H: 4.63, N: 1.85.)

### Surface area measurements

Volumetric gas sorption data for SZ-1, SZ-2 and SZ-3 were measured at 77 K using high-purity N_2_ gas in a liquid nitrogen bath and the detecting pressure ranges from 0 to 760 torr. CO_2_ sorption measurements were also performed at 195 K in a bath of dry ice and acetone. The pretreatment of three compounds were conducted as follows: all three compounds were soaked in aqueous saturated solution of LiCl, which was refreshed in every 24 h, and this process was kept for 3 days. Then, the solution was removed and the samples were washed several times by methanol. After decanting the methanol, the samples were dried under a dynamic vacuum (<10^−3^ torr) at room temperature overnight and then transferred into sample tubes. Before the gas adsorption measurement, the samples were activated using the ‘outgas' function of the surface area analyser for 4 h at 100 °C.

### Stability

Hydrolytic stability measurements for all three compounds were conducted. The samples were soaked in aqueous solutions at variable pHs (pH=1–14, solution of HNO_3_ or NaOH), sulfuric acid (3 mol l^−1^), and boiling water for one day. The powder X-ray diffraction results show that SZ-1, SZ-2 and SZ-3 are stable in boiling water and solution with the pH value ranging from 1 to 11. In addition, SZ-1 is stable even in a variety of fuming acid including aqua regia, oleum, concentrated HNO_3_ and HCl. About 100 mg of SZ-1 was used for checking the stability in fuming acid including aqua regia, oleum, concentrated HNO_3_ and HCl, for 12 h at room temperature. After the treatment, fuming acid was diluted and removed, and the solid sample was washed by water and ethanol for three times, individually, and further weighted after drying in an oven at 90 °C. For each case, <5% weight loss was found, demonstrating that SZ-1 is stable in the extreme acidic environment. Furthermore, large single crystals of SZ-1 can be still isolated from on the fuming acid-soaking samples. The crystal structures can be even successfully solved using the diffraction data collected on the oleum/aqua regia-soaked SZ-1 crystals, which are identical with the original structure. The stabilities of SZ-3 were checked using the same process with that of SZ-1 and the results show that they are similar to those of SZ-1, except in the case of oleum.

### Uranium sorption experiments

U(VI) sorption experiments have been performed at desired pH values. For each experiment, a total of 10 mg of SZ-1, SZ-2 and SZ-3 were added into a 50 ml container. Solution of U(VI) (10 p.p.m.) was added into the containers, with the solid–liquid ratio is 1 g l^−1^. The mixtures were kept on a shaking incubator for desired reaction times. The suspensions from the various reactions were filtrated and internal standard solution of indium was added for final analysis of uranium concentration using ICP-MS. U(VI) sorption experiments at desired pH values were also conducted, from 0.85 to 6.38.

X-ray absorption spectra were collected at the beamline 14 W1 of the Shanghai Synchrotron Radiation Facility with a Si(111) double crystal monochromator in transmission mode for the uranium L_3_-edge spectra. The electron beam energy of the storage ring was 3.5 GeV, and the maximum stored current was ∼210 mA. The uranium L_3_-edge EXAFS data were analysed using the standard procedures in Demeter^48^. Theoretical EXAFS data were calculated using FEFF 9.0 (ref. 49). Fitting procedure was performed on the *k*^3^-weighted FT-EXAFS from 2 to 10 Å^−1^. The amplitude reduction factor *S*_0_^2^ was fixed at 0.9 in EXAFS fits, and the shifts in the threshold energy Δ*E*_0_ were constrained to be the same value for all fitted shells.

Quantitative information was extracted by the EXAFS fitting, as shown in Fig. 7; Supplementary Table 9. Hydrated uranium atom exhibits two oxygen atoms for achieving uranyl unit and five water molecules to give a pentagonal bipyramid coordination geometry, with U-O distances are 1.77 and 2.42 Å, respectively. Interestingly, coordination geometry of the uranium absorbed in SZ-2 is quite similar to hydrated uranyl cations, except the coordination number is 4.4 instead of 5. This is likely induced by confinement effect of the channels of the framework.

### Quantum mechanics and molecular dynamics simulations

Force Parameterization of SZ-2 was obtained from quantum mechanics calculations. First-principle calculations based on the density functional theory were performed using the generalized gradient approximation (GGA) with the Becke three parameters hybrid exchange-correlation functional^50^,^51^ implemented in the Gaussian 09 program^52^. The smallest unrepeatable unit extracted from the experimentally obtained crystal structure was chosen as the computational model, as seen in Supplementary Fig. 26. The standard Gaussian-type basis sets 6-31G* (ref. 53) was used for C, O, F, P and H atoms. To include the scalar relativistic effects, the relativistic effective core potential ECP28MWB (ref. 54) and its corresponding (8s7p6d2f1g)/[6s5p3d2f1g] valence basis set^55^ were selected for Zr atoms. The atomic configuration of the computational model was kept the same as the experimentally determined structure and hence a single-point energy calculation was performed. On the basis of the ground state electron density obtained by the density functional theory, the atomic charges fit to the electrostatic potential at points selected (ESP charge) according to the CHelp scheme^56^ (using the Chirlian–Francl model) were then deduced. To ensure the accuracy, 10-concentric layers of points were used for each atom and >7,000 points in total were used for fitting atomic charges. These atomic charges were then used for the following classical MD simulations.

The initial configuration of SZ-2 was obtained from our X-ray crystal structure with a size of (6.616 × 6.616 × 4.749 nm^3^), containing 6,528 atoms (Supplementary Fig. 25). The SZ-2 force field parameters were obtained from our quantum mechanics calculations. The parameters of uranyl cation were adopted from a previous literature, in which the hydration behaviours of the equatorial plane of the uranyl cation have been elaborately studied^57^. System-1 and system-2 are only differing in the relative position of uranyl cation to the facets of SZ-2, to mimic the adsorption of uranyl cation into the SZ-2 from two different directions: the top (Supplementary Fig. 26a,b) and the side direction (Supplementary Fig. 26c,d). Initially, the SZ-2 were solvated into two different rectangular water boxes with the size of (6.616 × 6.616 × 10.0 nm^3^) and (6.616 × 12.616 × 4.749 nm^3^), then 384 Na^+^ were added to neutralize both systems. After two 1 ns NVT individual equilibriums, a uranyl cation was then introduced into two water boxes, with a distance between any heavy atoms of SZ-2 at least 1.5 nm. Meanwhile, 2 Na^+^ were removed to balance the charges of uranyl cation (that is, to neutralize the simulation systems again; in other words, both system-1 and system-2 contain a framework fragment of SZ-2, a uranyl cation and 382 Na^+^). While, system-1 containing 12401 water molecules, the system-2 containing 11,454 water molecules, respectively. This fully solvated complex was then simulated with molecular dynamics simulations, which are widely used in the studies of biomolecules^58^,^59^,^60^,^61^,^62^,^63^,^64^ and nanomaterials^65^,^66^,^67^,^68^,^69^,^70^.

The MD simulations were performed with the software package GROMACS (version 5.0.2)^71^. The VMD software^72^ was used to analyse and visualize the simulation results. The TIP3P water model^73^ was used for water molecules. During the simulation, temperature was maintained at 300 K by using *v*-rescale thermostat and the volume of the simulation box also remained constant (NVT)^74^. Periodic boundary conditions were applied in all directions. The SZ-2 was frozen throughout the simulation process. The long-range electrostatic interactions were treated with the PME method^75^, and the van der Waals (vdW) interactions were calculated with a cutoff distance of 1.2 nm. All solute bonds were maintained constant at their equilibrium values with the LINCS algorithm^76^, and water geometry was also constrained using the SETTLE algorithm^77^. During the production runs, a time step of 2.0 fs was used, and data were collected every 5 ps. For both the system-1 and system-2, six independent of 100 ns repeats were performed. The total aggregated simulation time was >1.2 μs.

### Data availability

The X-ray crystallographic coordinates for structures reported in this study are provided as cif files in Supplementary Data 1 and have been deposited at the Cambridge Crystallographic Data Centre, under deposition numbers 1450124, 1450121, 1531836, 1450122 and 1450123. These data can be obtained free of charge from The Cambridge Crystallographic Data Centre via HYPERLINK http://www.ccdc.cam.ac.uk/data_request/cif www.ccdc.cam.ac.uk/data_request/cif.

## Additional information

**How to cite this article:** Zheng, T. *et al*. Overcoming the crystallization and designability issues in the ultrastable zirconium phosphonate framework system. *Nat. Commun.* **8,** 15369 doi: 10.1038/ncomms15369 (2017).

**Publisher's note:** Springer Nature remains neutral with regard to jurisdictional claims in published maps and institutional affiliations.

## Supplementary Material

Supplementary Information Supplementary Figures, Supplementary Tables and Supplementary References

Supplementary Data 1 Cif files for SZ-1, SZ-2, SZ-3, Tppm ligand and its ester precursor.
