# Supplementary material for: Overcoming the crystallization and designability issues in the ultrastable zirconium phosphonate framework system
Source: Nat Commun. 2017 May 30;8:15369. doi: 10.1038/ncomms15369 (PMC5459948; doi:10.1038/ncomms15369)
Supplement: Supplementary Information — Supplementary Figures, Supplementary Tables and Supplementary References [file ncomms15369-s1.pdf]

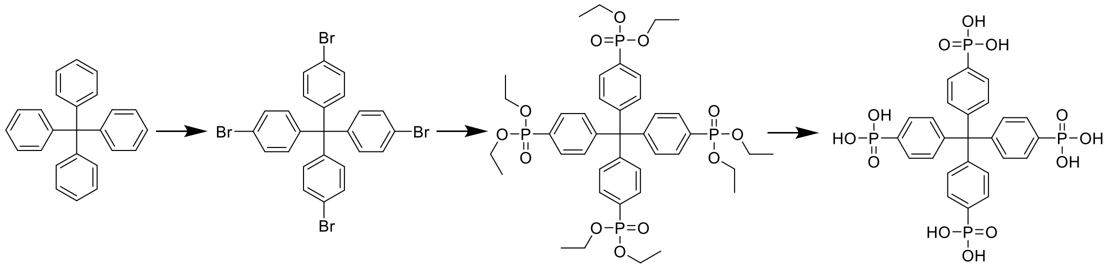

A B $TppmH_8$

Supplementary Figure 1: Synthesis route for $TppmH_8$.

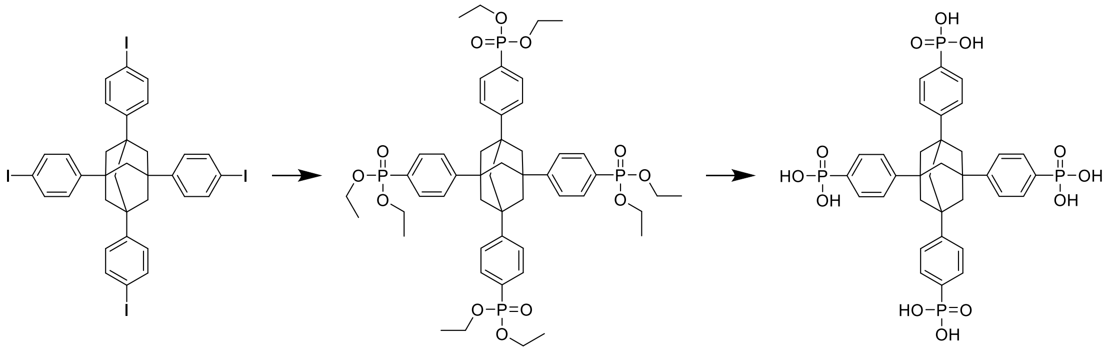

C $TppaH_8$

Supplementary Figure 2: Synthesis route for $TppaH_8$.

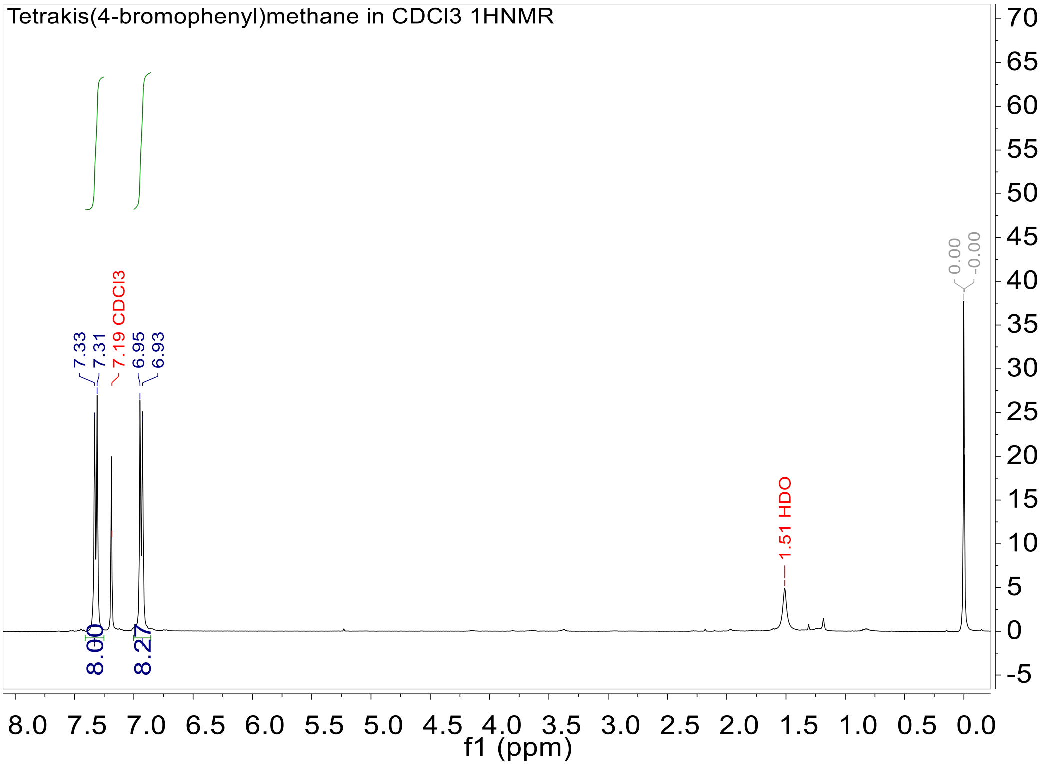

Supplementary Figure 3: ^{1}HNMR spectrum of Tetrakis(4-bromophenyl)methane (A). Tetrakis(4-bromophenyl)methane (A)[1] :Tetraphenylmethane (15.00 g, 46.8 mmol) was placed into a 100 mL round-bottom flask under continuous stirring and bromine (10.00 mL, 330.8 mmol) was added in a period of 1 h. The resulting dark-brown slurry was stirred for an additional 8 h and then transferred into an ethanol (400 mL) solution. The solution was cooled down to -78 ^{o}C using liquid nitrogen. The yellow solid was filtered and washed with saturated aqueous $NaHSO_3$ solution (120 mL × 3). The final yellow products were dried at 90 ^{o}C to remove solvent (28.87 g, 45.39 mmol) in 97% yield. ^{1}H-NMR (400 MHz, CDCl3): δ 7.33-7.31 (8H, C_6H_2), 6.956-6.93 (8H, C_6H_2).

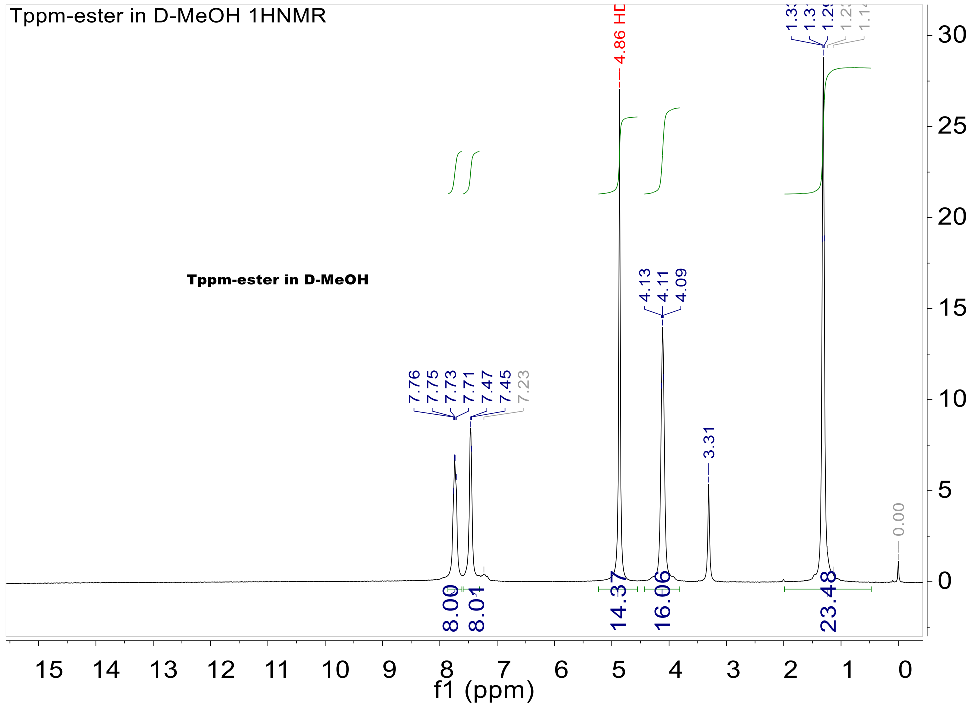

Supplementary Figure 4: ^{1}HNMR spectrum of Tetrakis[4-(dietoxyphosphoryl)phenyl]methane (**B**).[2] Compound **A** (10.66 g, 16.8 mmol) and 1,3-diisopropylbenzene (40 mL) were placed into a three-neck round-bottom flask and heated to 180 °C within 20 min under nitrogen atmosphere. Nickel(II) bromide (2.1 g, 9.6 mmol) was added into the mixture after half hour. After 10 min, triethyl phosphite (20 mL, 116 mmol) was slowly added in a period of 5 h. Additional nickel bromide (1.1 g, 5 mmol) and triethyl phosphite (10 mL, 58 mmol) were added into the mixture, after 24 h. After additional 24 h, the reaction mixture was cooled down to room temperature. The mixture was extracted with water and acetate ester for three times to remove Ni^{2+}. The organic phase was collected and after the removal of acetate ester, triethyl phosphite, and 1,3-diisopropylbenzene in a vacuum, oil-like solid was obtained. The solid was dissolved in 100 mL acetate ester and transferred into a refrigerator (-40 °C) for a night. White solid was obtained by filtration, washed with ethyl ether, and dried under vacuum, with the yield of 64.3% (9.36 g, 10.8 mmol). ^{1}H-NMR(400 MHz, CD3OD): δ 7.76-7.71 (8H,C_6H_2), 7.47-7.45 (8H, C_6H_2), 4.13-4.09 (16H, P-O-CH_2-C), 4.15-3.99 (8H, PO-CH_2-C), 1.33-1.29 (24H, PO-C-CH_3) (Figure S2). Single crystals of compound **B** denoted as

Tppm-ester were obtained by evaporation of ethanol solution of **B** at room temperature. The crystallographic results are listed in Supplementary Table 1.

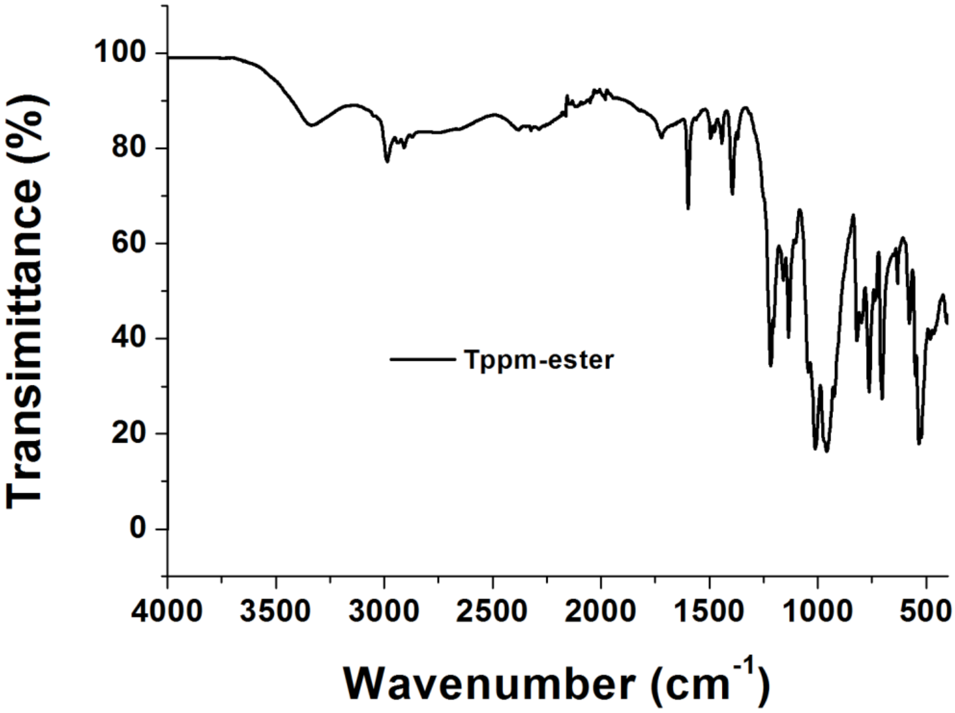

Supplementary Figure 5: IR spectrum of Tetrakis[4-(dietoxyphosphoryl)phenyl]methane (**B**).

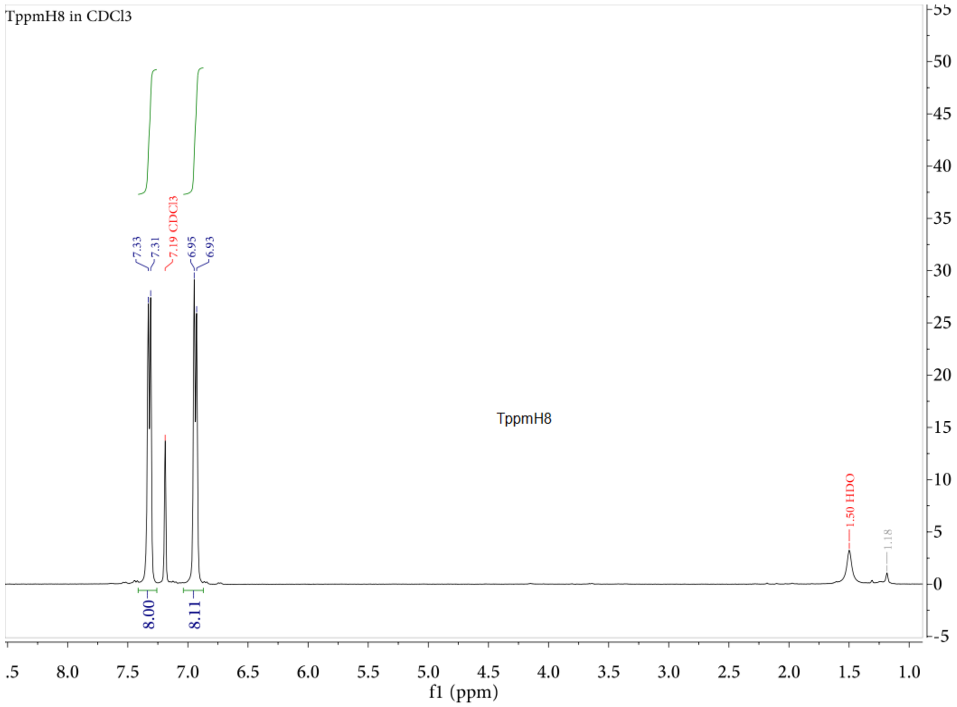

Supplementary Figure 6: ^{1}HNMR of Tetrakis[4-(dihyroxyphosphoryl)phenyl]methane ($TppmH_8$).

Tetrakis[4-(dihyroxyphosphoryl)phenyl]methane ($TppmH_8$) [2]. Compound **B** (5 g, 5.78 mmol) was dissolved in dichloromethane (30 mL) and trimethylsillyl bromide (9 mL) in a round-bottom flask. The green solution was stirred for 1 day at room temperature and then evaporated under vacuum. Methanol (85 mL) was added into the flask and evaporated for three times. The obtained solid was dissolved in water and the pH of solution was adjusted to 7 using a solution of sodium hydroxide (1 mol/L) and active carbon was then added. Filtrate was collected and acidified with concentrated hydrochloric acid (36%). White solid of $TppmH_8$ was obtained by filtration with the yield of 95.2% (3.52 g, 5.50 mmol). Partially hydrolyzed products of $TppmH_8$ can also be obtained by refluxing compound **B** in concentrated hydrochloric acid (36%) overnight. ^{1}H-NMR (400 MHz, $CDCl_3$): δ 7.33-7.31 (8H,C_6H_2), 6.95-6.93 (8H, C_6H_2), (Figure S4).

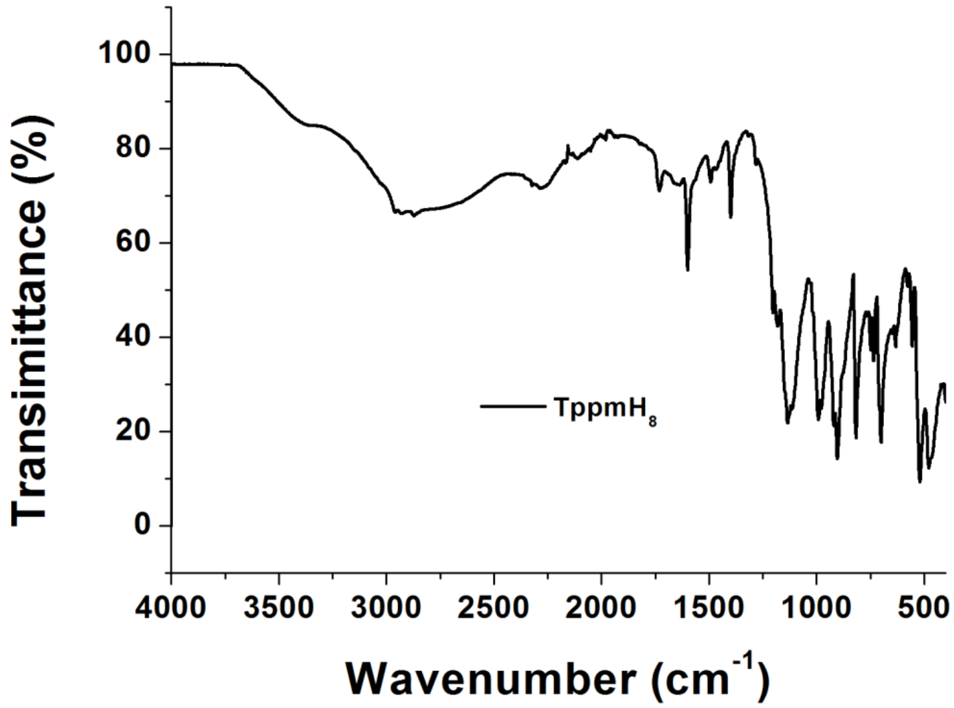

Supplementary Figure 7: IR of Tetrakis[4-(dihyroxyphosphoryl)phenyl]methane ($TppmH_8$).

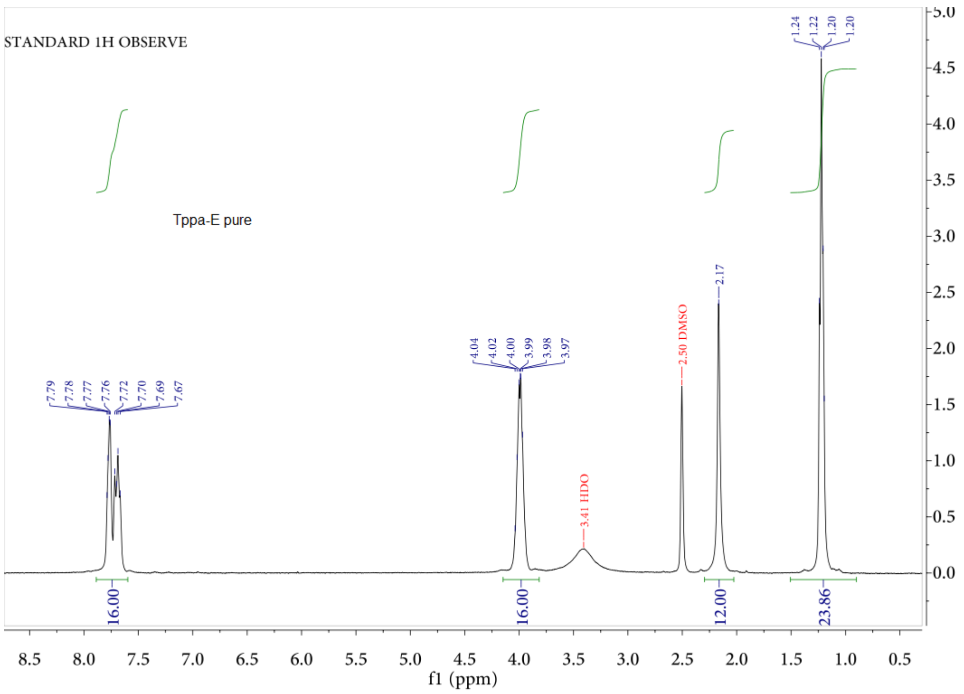

Supplementary Figure 8: ^{1}HNMR of Tetrakis[4-(diethoxyphosphoryl)phenyl] adamantane (Tppa-Ester, **C**).[3, 4] Tetrakis(4-iodophenyl)adamantane (2.28 mmol) was placed into a roundbottom flask with $NiBr_2$ anhydrous (20%), 1,3-diisopropylbenzene (80 ml) and fitted with a condenser and a dropping funnel. $P(OEt)_3$ (3 ml, 16.95 mmol) was placed in to the dropping funnel with diisopropylbenzene (6 ml). The solution was brought to reflux and the $P(OEt)_3$ solution added slowly over 8 h, followed by refluxing for 24 h. Upon cooling, a precipitate formed that was subsequently filtered, washed with ether and identified as the phosphonate diethyl ester of **C** (1.9g,85%). ^{1}H NMR (CDCl3), δ 7.77 (m, 2H), 7.59 (m, 2H), 4.13 (m, 4H), 2.21(s, 3H), 1.33(t, 6H) ppm.

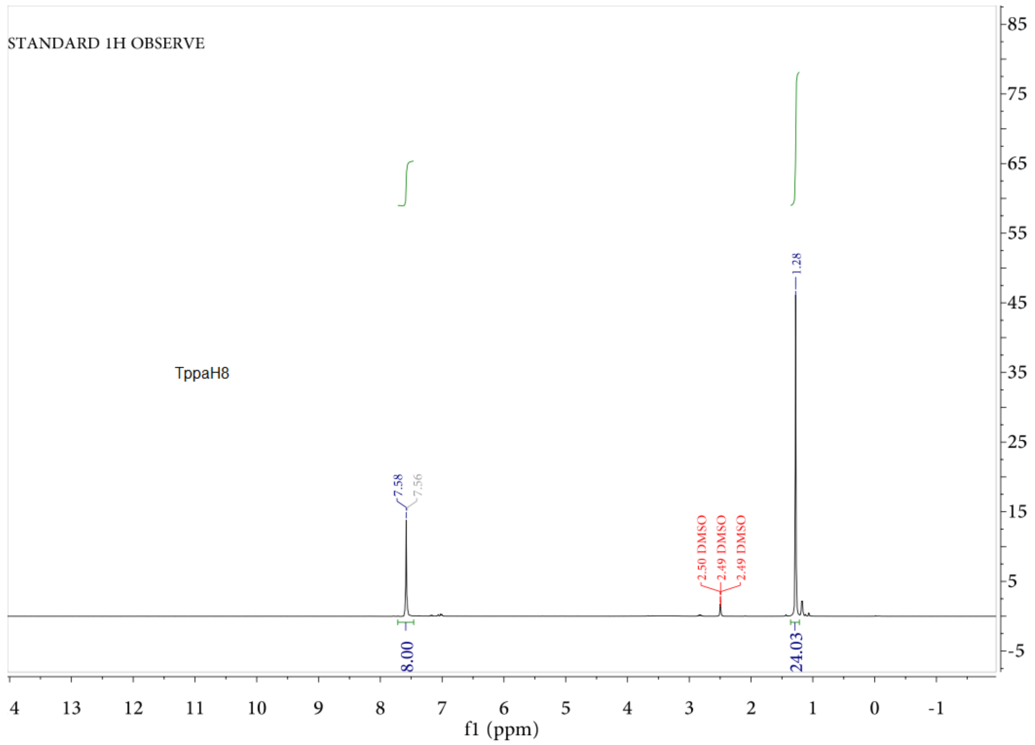

Supplementary Figure 9: ^{1}HNMR of Tetrakis[4-(dihyroxyphosphoryl)phenyl]methane ($TppaH_8$). **C** (0.985g, 1.0 mmol) was placed into a round-bottom flask with concentrated HCl (20 ml) and refluxed for 48 h. It was then filtered to yield a light beige solid, which was treated with MeOH and the insoluble material removed by filtration. The MeOH was then removed by vacuum to give **$TppaH_8$** as a white solid. (0.62 g, 82%).

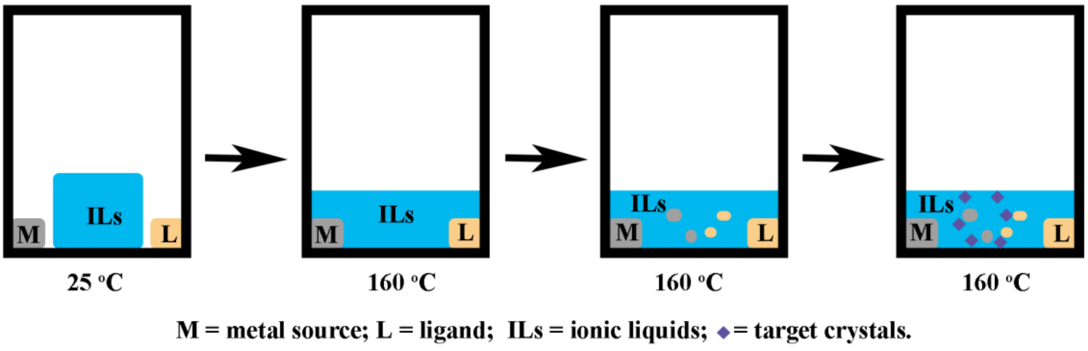

Supplementary Figure 10: Diffusion method for synthesis of pure **SZ-2** material.

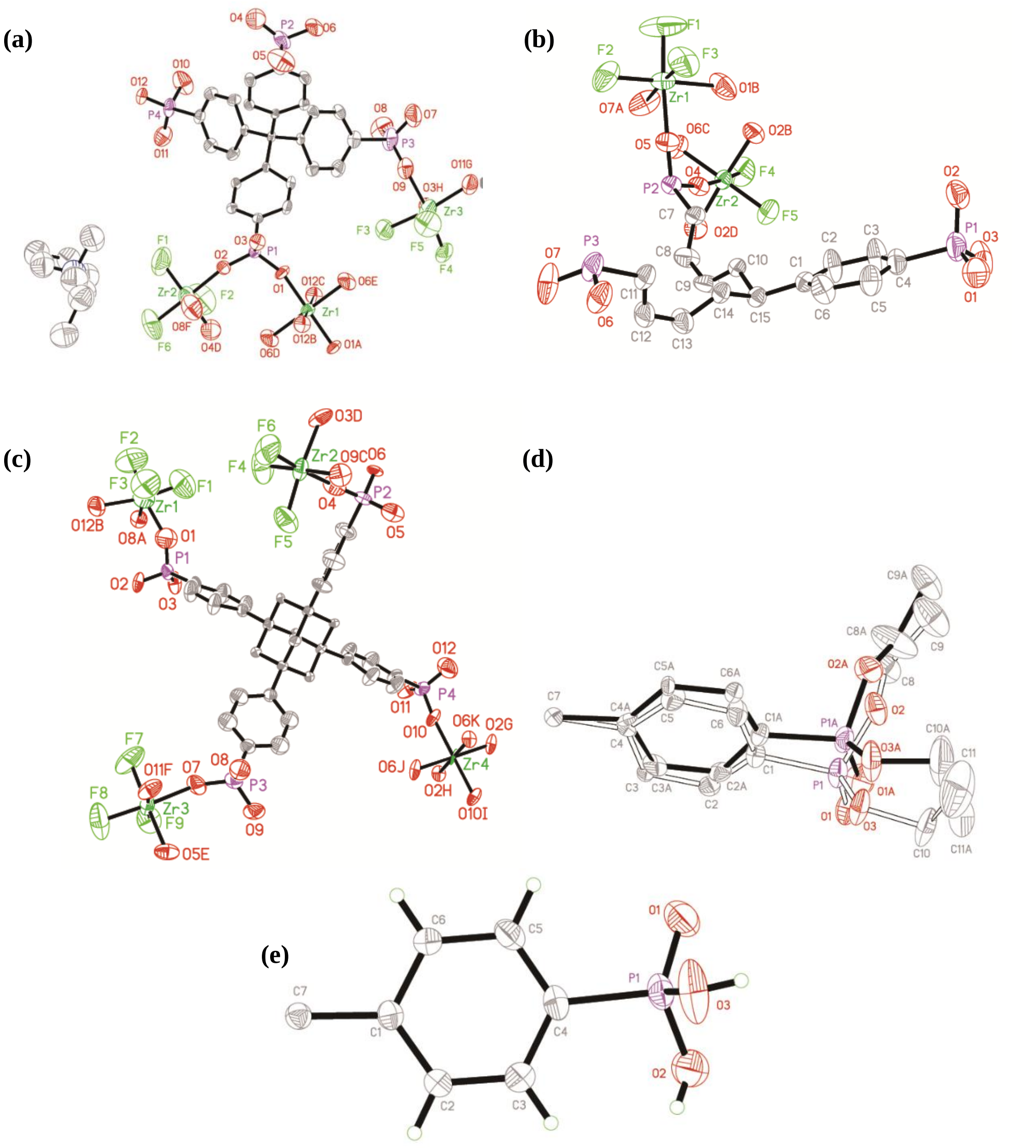

Supplementary Figure 11: Asymmetric unit of **SZ-1** (a), **SZ-2** (b), **SZ-3** (c), Tppm-ester (d), and $TppmH_8$ (e) with atomic labelling scheme at 50% probability, and all hydrogen atoms are omitted for clarity. There are two parts in both **SZ-1** and Tppm-ester, and the other part is omitted for clarity.

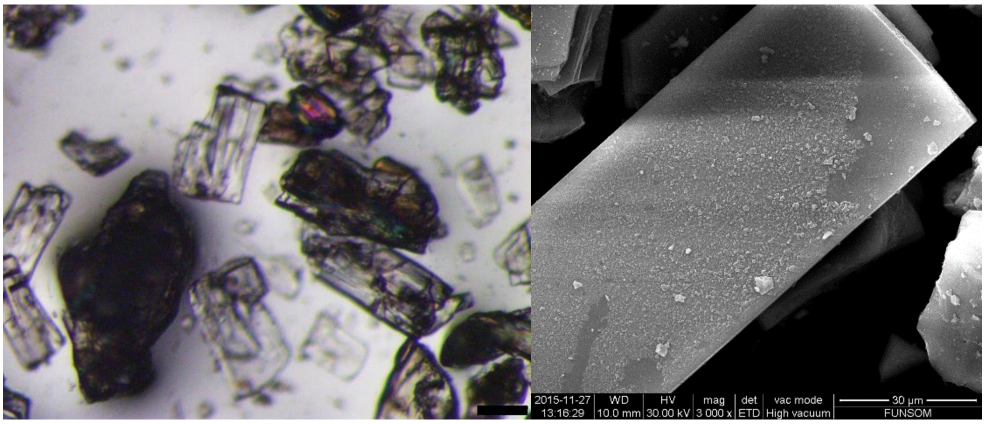

Supplementary Figure 12: Optical microscope (left, the scale bar is 100 µm) and SEM (right) images of **SZ-1.**

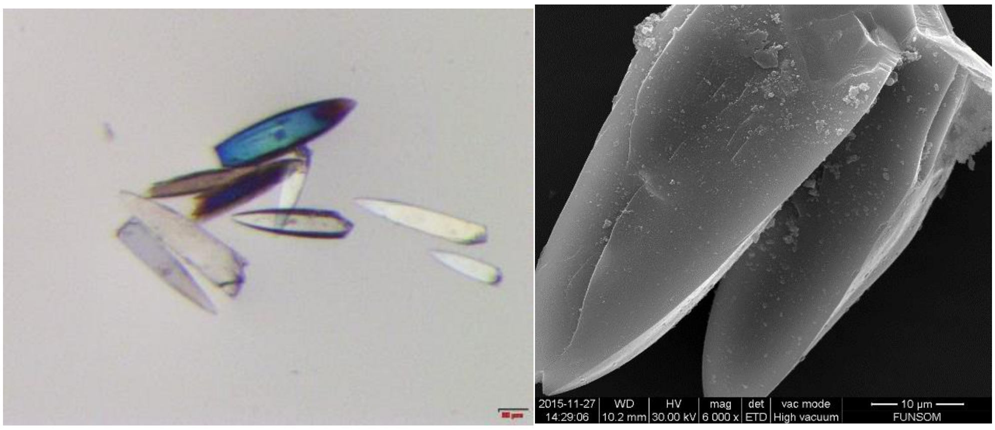

Supplementary Figure 13: Optical microscope (left, the scale bar is 50 µm) and SEM (right) images of **SZ-2**.

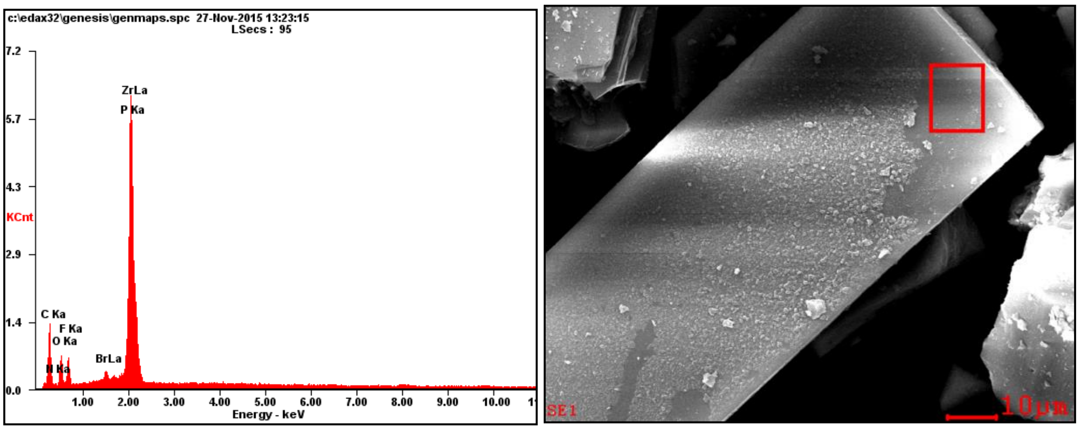

Supplementary Figure 14: EDS spectrum collected on the marked area of **SZ-1**.

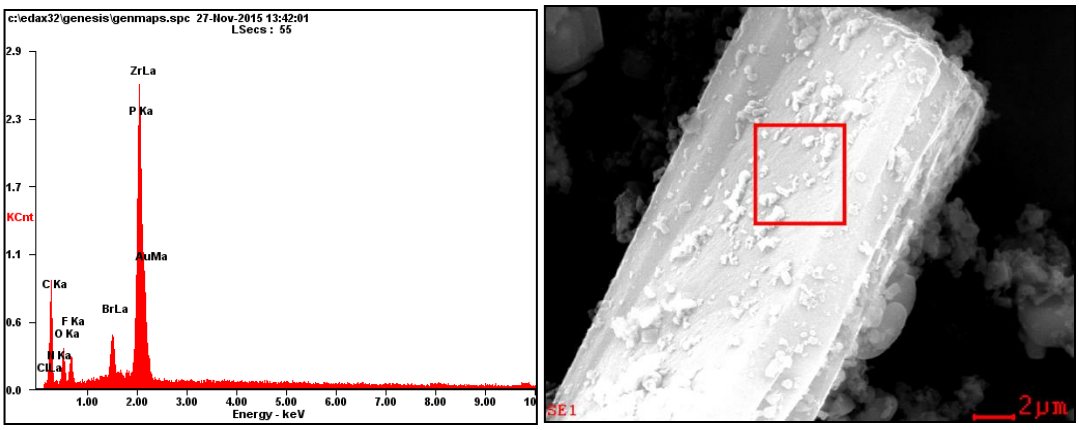

Supplementary Figure 15: EDS spectrum collected on the marked area of **SZ-2**.

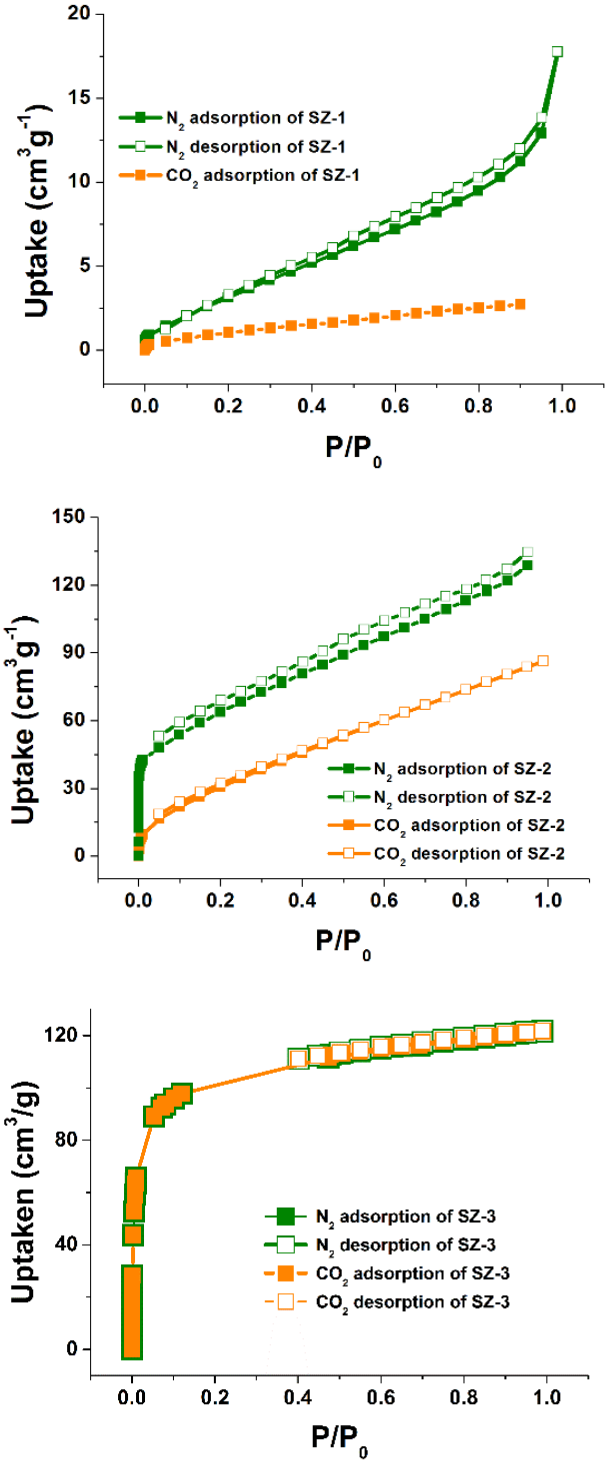

Supplementary Figure 16: N_2 and CO_2 sorption isotherms of **SZ-1**, **SZ-2**, and **SZ-3** . **SZ-1** exhibits moderate porosity with a N_2 BET and Langmuir surface area of 10.2 and 16.5 m^{2}g^{-1}, and a CO_2 BET and Langmuir surface area of 5.27 and 16.5 m^{2}g^{-1}, respectively respectively, likely because of the difficulty of exchanging C_4mpyr$^+$ with other cations (Figure S12a) even in concentrated acid. While BET and Langmuir surface area of **SZ-2** is 225 and 375 m^{2}g^{-1}, respectively, calculated using the N_2

adsorption data. While using the CO_2 data, the BET and Langmuir surface area of **SZ-2** is measured to be slightly lower at 161 and 331 m^2g^{-1}, respectively. The BET and Langmuir surface area of **SZ-3** is 572 and 695 m^2g^{-1}, respectively, calculated using the N_2 adsorption data, however, the BET and Langmuir surface area of **SZ-3** measured by CO_2 to be slightly lower at 520 and 673 m^2g^{-1}, respectively.

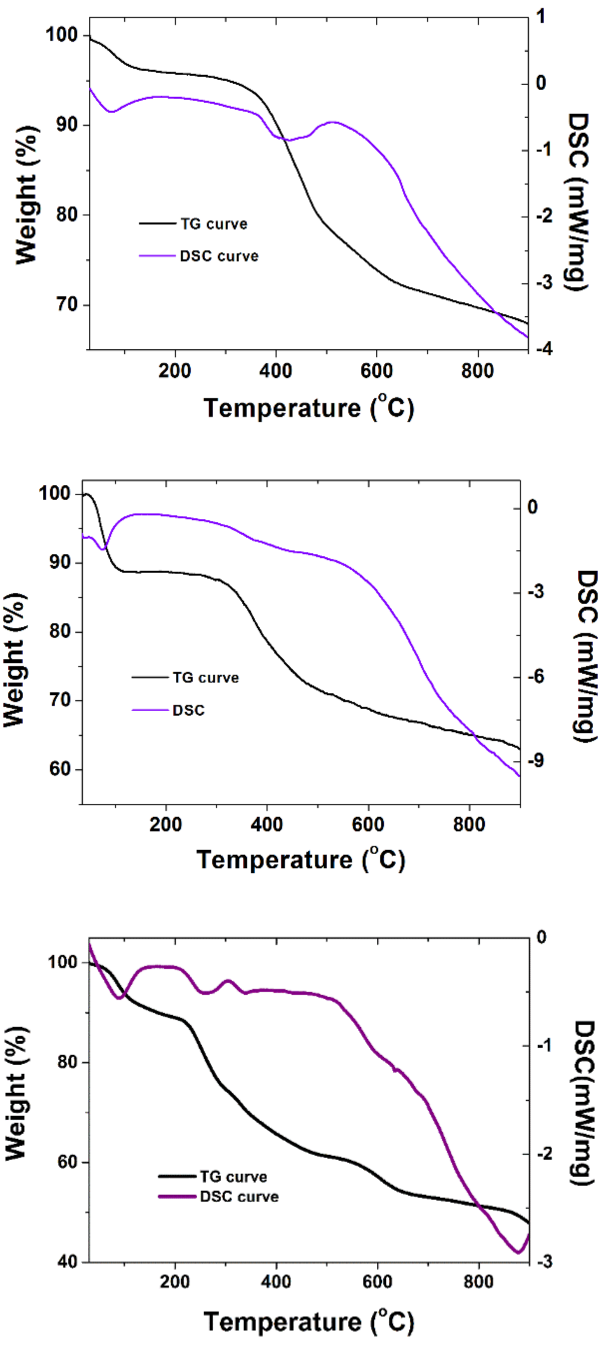

Supplementary Figure 17: TG-DSC curves of compounds **SZ-1**, **SZ-2** and **SZ-3**. Thermogravimetric analyses were carried out on a NETZSCH STA 449F3 instrument in the range of 30–900 °C under a nitrogen flow at a heating rate of 10 °C/ min. Variable-temperature powder X-ray diffraction (VT-PXRD) were carried out on a Bruker D8 Advance diffractometer from 25 to 600 °C (Figure S13). Two weight loss steps were observed in **SZ-1**. The first 4.06% (cal. 4.03%) weight loss at 180 °C is attributed to the loss of two and half water molecules. The second one is attributed to

the framework collapse of **SZ-1**, consistent with the result of VT-PXRD measurement (Figure S14). For **SZ-2**, the first weight loss of 11.29% (cal. 11.75%) at 100 ^{o}C is due to the loss of six lattice water molecules filled in the channels of **SZ-2**, and it is stable up to ca. 300 ^{o}C, with the second weight loss originating from the framework collapse. For **SZ-3**, the first weight loss of 7.12% (cal. 7.42 %) at 110 ^{o}C is due to the loss of six and half lattice water molecules and it collapse at ca. 210 ^{o}C.

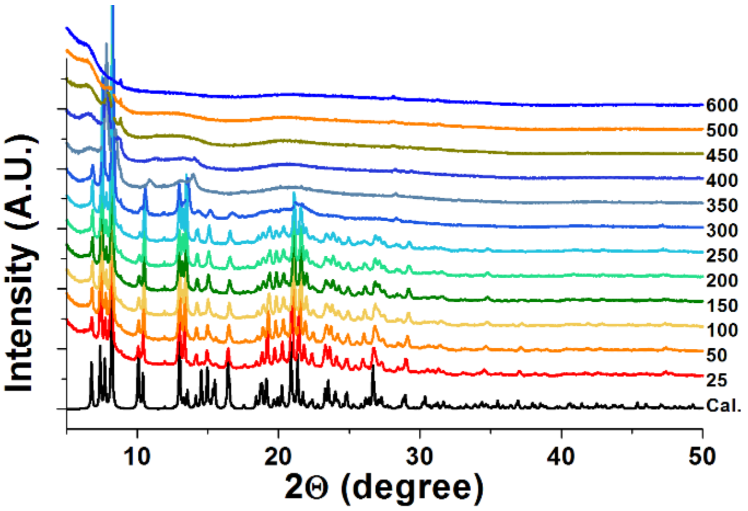

Supplementary Figure 18: VT-PXRD patterns of **SZ-1** showing that **SZ-1** starts to collapse at ca. 300 ^{o}C.

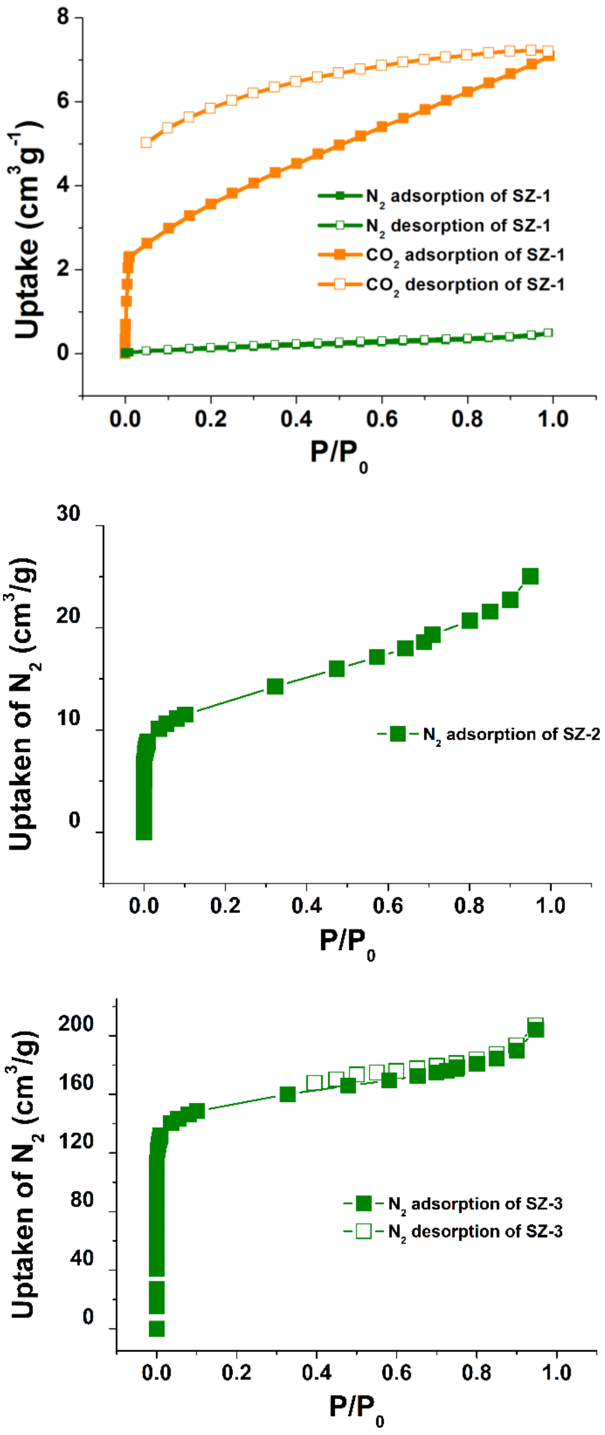

Supplementary Figure 19: N_2 sorption isotherms of **SZ-1**, **SZ-2**, and **SZ-3** after chemical stability test (aqua regia). **SZ-1** soaked in aqua regia exhibits porosity with a N_2 BET and Langmuir surface area of 0.54 and 1.23 m^2g^{-1}, respectively, while CO_2 BET and Langmuir surface area is 16.28 and 27.56 m^2g^{-1}, respectively (right). N_2 BET and Langmuir surface area of **SZ-2** decreased to 45.5 and 72.9 m^2g^{-1}, respectively. N_2 BET and Langmuir surface area of **SZ-3** is 594 and 732 m^2g^{-1}, respectively.

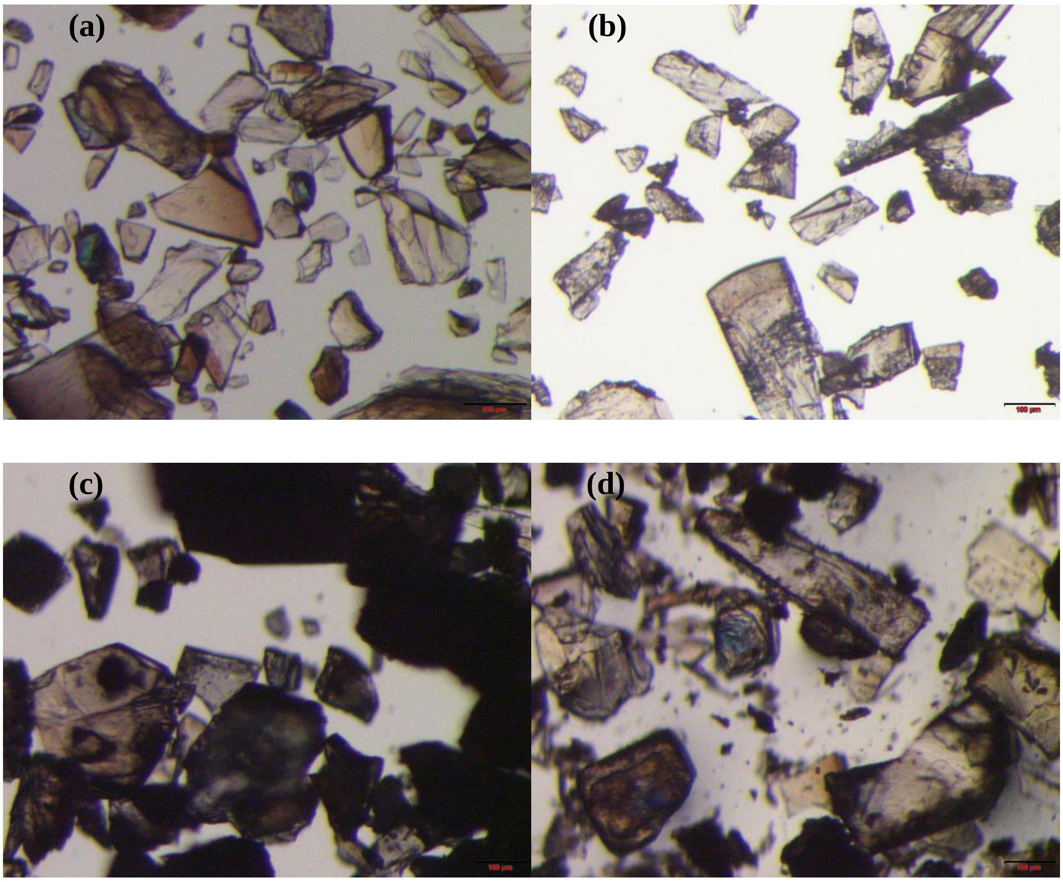

Supplementary Figure 20: Optical microscope images of **SZ-1** after soaked in aqua regia (a), oleum (b), concentrated HNO_3 (c), and concentrated HCl (d) (the scale bar is 100 μm), showing that the crystallinity of **SZ-1** is preserved, which is confirmed by powder (see main text) and single-crystal X-ray analysis on the fuming acid-soaking samples.

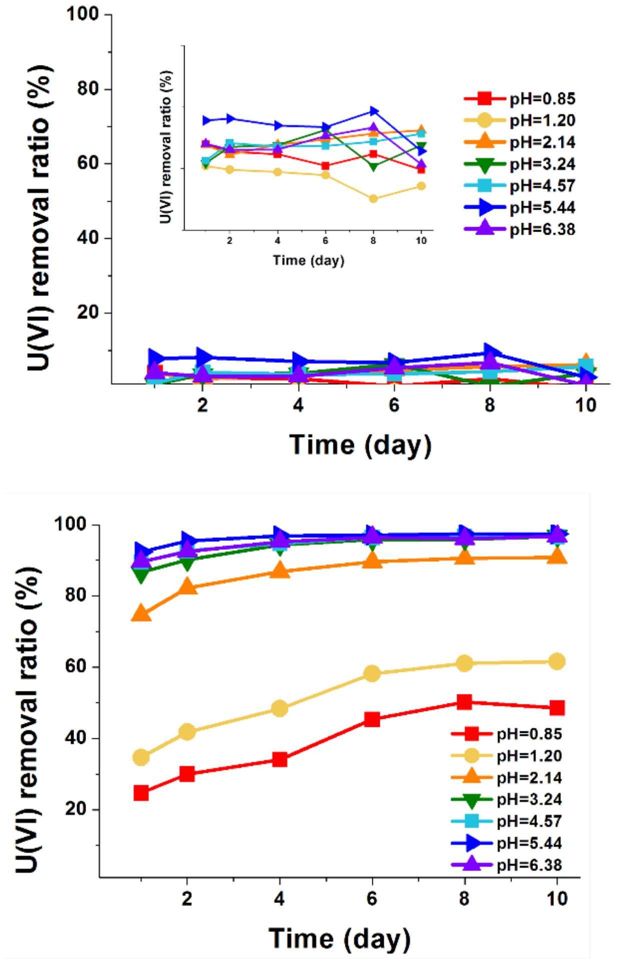

Supplementary Figure 21: Uranyl(VI) ion uptake kinetics for **SZ-1** (left) and **SZ-2** (right) in aqueous solutions with different pH values. The uptake amount using SZ-1 is significantly lower than that of the mixed **SZ-1** and **SZ-2** sample, indicating that **SZ-2** is mainly responsible for the high uranium uptake ability of the mixed sample (inset: Figure with a smaller y-axis).

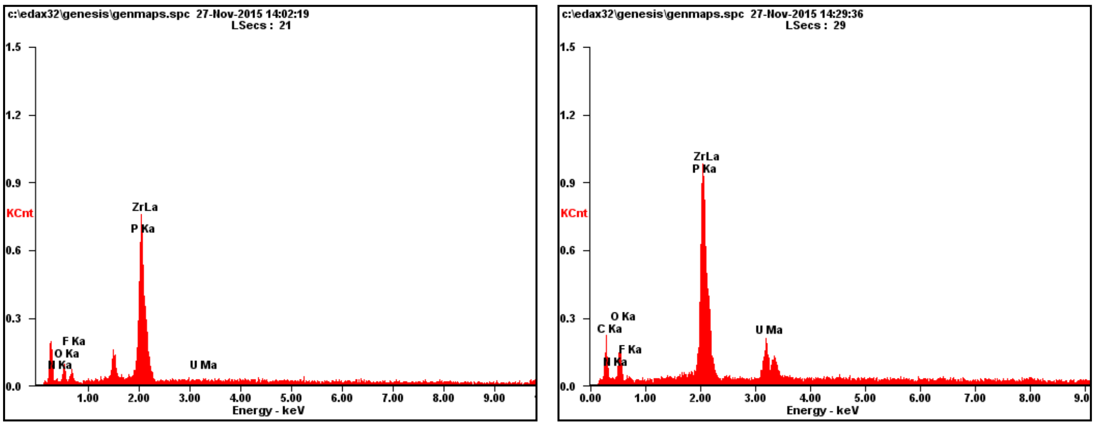

Supplementary Figure 22: EDS patterns of **SZ-2** (left) and **SZ-2-U** (right).

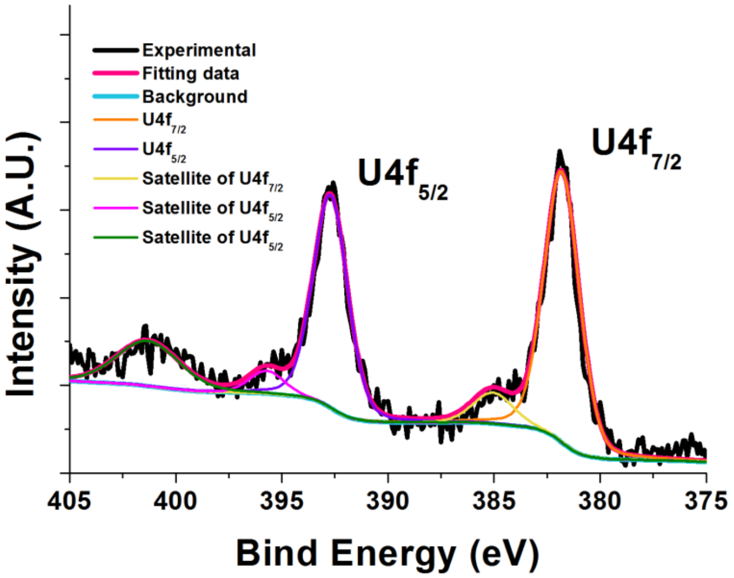

Supplementary Figure 23: The U4f XPS spectrum of **SZ-2-U**.

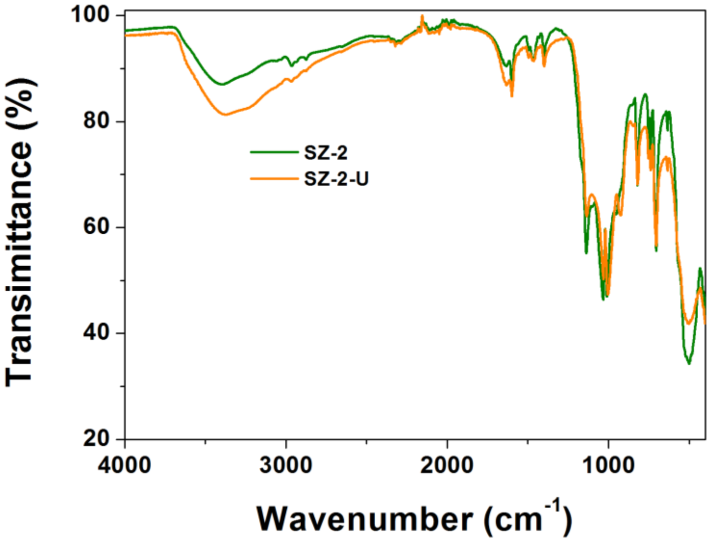

Supplementary Figure 24: IR spectra of **SZ-2** and **SZ-2-U**. The IR spectra of **SZ-2** and **SZ-2-U** in high-wavenumber region are dominated by the bands assigned for O-H and C-H stretches. The intensity of **SZ-2-U** (broad medium peaks around 3382 cm^{-1}) is stronger than the that of **SZ-2**, which may be due to additional hydrogen bonds formed after hydrated uranyl ions were exchanged into the channels of **SZ-2**. The peaks at 1138, 951, and 500 cm^{-1} are assigned to phosphonate group, which also effected by hydrated uranium, and the strength are all decreased consistent with the theoretical calculation (see below). The peak of 927 cm^{-1} is attributed to O=U=O

antisymmetric stretch.

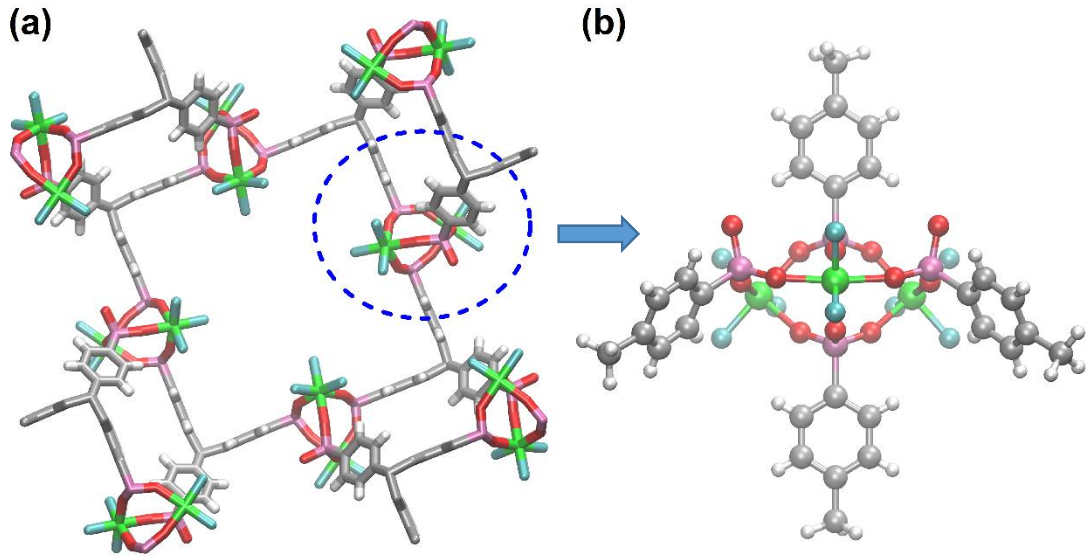

Supplementary Figure 25: (a) Schematic diagram of the **SZ-2** unit cell. The dot circle line shows the smallest unrepeatable unit. (b) The smallest unrepeatable unit extracted from the **SZ-2** unit cell which was chosen as the DFT computational model. The green, cyan, red, purple, gray, and white balls indicate the Zr, F, O, P, C, and H atoms. The dangling carbon atoms of the fault surfaces were terminated by H atoms. Four negative charges were added to this model during DFT calculations to match the formal valence state of the whole system.

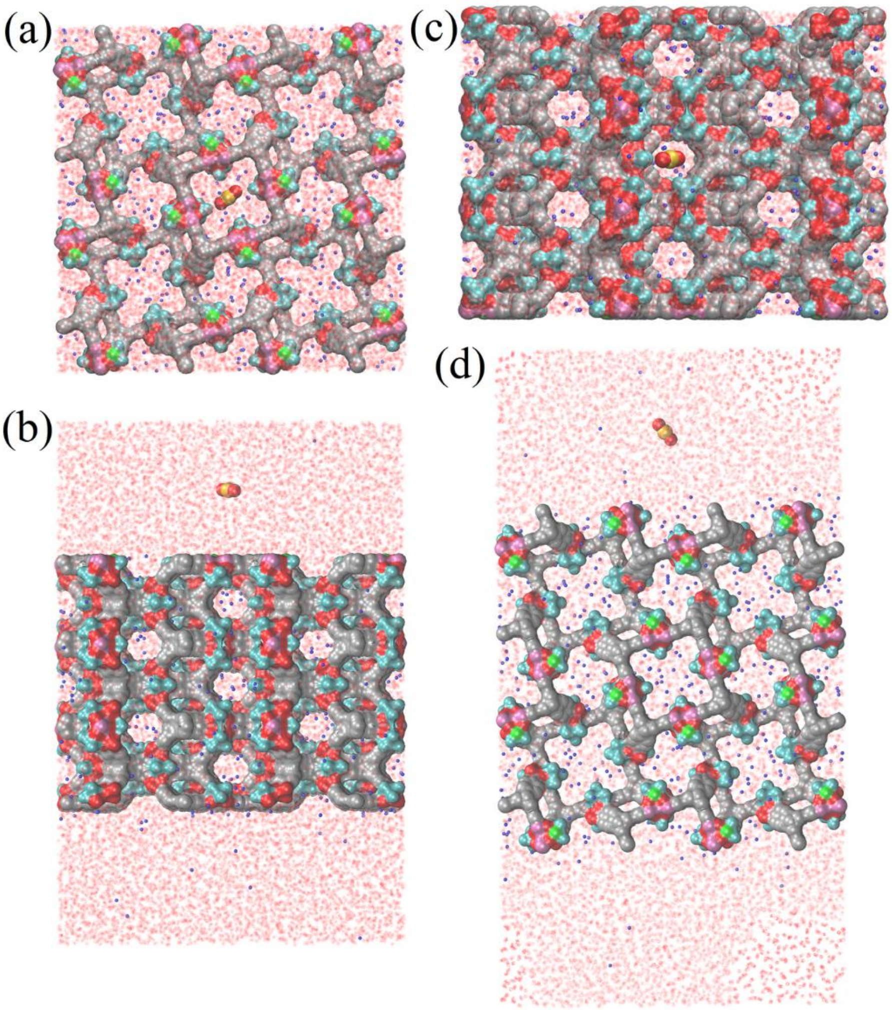

Supplementary Figure 26: The top view and the side view of the initial configurations of system-1 (a and b) and system-2 (c and d). The color scheme for **SZ-2** is the same as Figure 4. The uranyl cation, Na^+, and water molecules are shown as vdW ball, blue dots, and thick transparent bonds representation, respectively. U is coloured in golden.

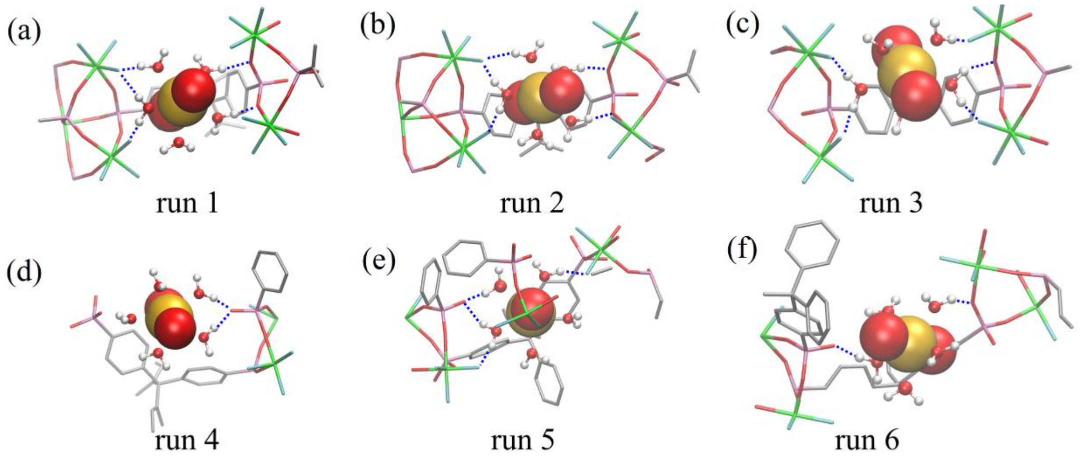

Supplementary Figure 27: (a-f) The final snapshots of six independent runs (at t = 100 ns) for system-1 (uranyl cation approaching from the top). The blue dash lines stand for the hydrogen bonds formed between the equatorial water molecules of uranyl cation and the dangling hydrogen bond acceptors (including F and O that originally bind to Zr and P) in **SZ-2**.

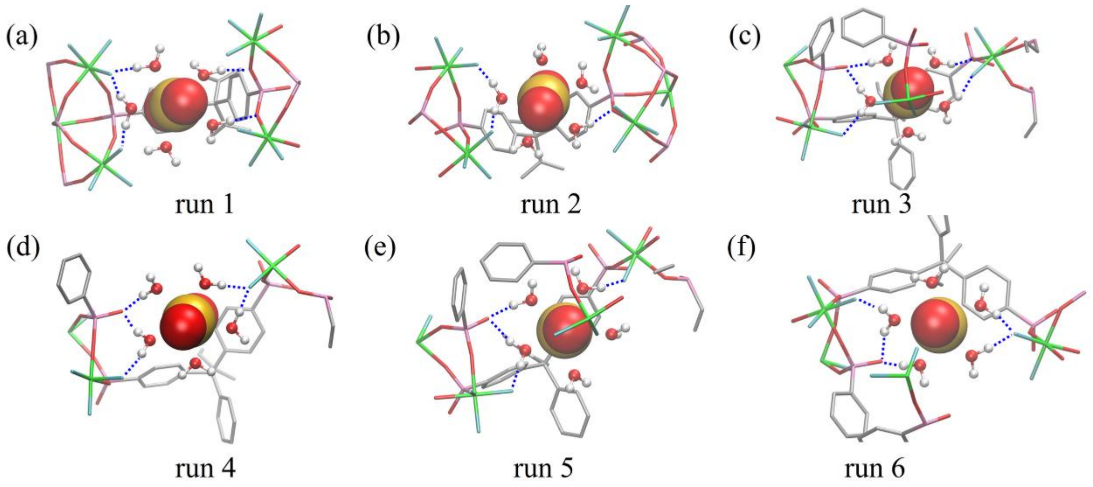

Supplementary Figure 28: (a-f) The final snapshots of six independent runs (at t = 100 ns) for system-2 (uranyl cation approaching from the side). The blue dash lines stand for the hydrogen bonds formed between the equatorial water molecules of uranyl cation and the dangling hydrogen bond acceptors (including F and O that originally bind to Zr and P) in **SZ-2**.

Supplementary Table 1. Crystal data and refinement details for **SZ-1**, **SZ-2**, **SZ-3**, **Tppm-ester,** and **Tppm**H_8.

	**SZ-1**	**SZ-2**	**SZ-3**	**Tppm-ester**	**Tppm**H_8
Formula	$C_{34}H_{36}F_6NO_{12}P_4Zr_{2.5}$	$C_{25}H_{16}F_8O_{12}P_4Zr_3$	$C_{68}H_{48}F_{18}O_{24}P_8Zr_7$	$C_{41}O_{16}P_4$	$C_{25}H_{24}O_{12}P_4$
CCDC No.	1450124	1450121	1531836	1450122	1450123
M	1145.78	1057.92	2477.37	872.29	640.32
Crystal system	Orthorhombic	Tetragonal	Monoclinic	Tetragonal	Tetragonal
Space group	*Pbca*	*I*4/*m*	*P*2(1)/*n*	*I*4(1)/*a*	$I\bar{4}$
a / Å	23.9163(17)	33.081(5)	16.9609(13)	24.905(3)	13.2646(7)
b / Å	13.5859(10)	33.081(5)	26.425(2) A	24.905(3)	13.2646(7)
c / Å	26.106(2)	15.832(3)	16.9629(13)	7.5896(10)	7.5238(5)
α / °	90	90	90	90	90
β / °	90	90	117.718(2)	90	90
γ / °	90	90	90	90	90
V /Å^3	8482.5(11)	17326(6)	6730.2(9)	4707.7(13)	1323.82(17)
Z	8	8	2	4	2
ρ_{calcd} / gcm^{-3}	1.577	0.811	1.222	1.231	1.606
F (000)	3932	4112	2420	1736	660
(Mo-K) /mm^{-1}	0.877	0.469	0.686	0.224	0.352
GooF on F^2	1.042	0.973	1.053	1.059	1.075
R_1,	0.0869,	0.0674,	0.1085,	0.0763,	0.0432,
wR_2 [I>2σ(I)]	0.2452	0.2165	0.3076	0.2121	0.1028
R_1,	0.1266,	0.0994,	0.1317,	0.0986,	0.0516,
wR_2 (all data)a	0.2697	0.2456	0.3315	0.2332	0.1218
(Δρ)max,	1.565,	1.325,	2.562,	0.580,	0.415,
(Δρ)min / eÅ^{-3}	–0.779	–0.613	-2.991	–0.296	-0.554

$^aR_1 = \Sigma||F_o|–|F_c||/\Sigma|F_o|$. $wR_2=[\Sigma w(F_o2-F_c^2)^2/\Sigma w(F_o^2)^2]^{1/2}$

Supplementary Table 2. Selected bond distances (Å) and angles (^{o}) in **SZ-1**.

Zr1-O1	1.951(9)	Zr3-O9	2.041(9)
Zr1-O1A	1.951(9)	Zr3-O11G	2.162(15)
Zr1-O12B	2.042(11)	Zr3-O3H	2.147(14)
Zr1-O12C	2.042(11)	P1-O3	1.505(9)
Zr1-O6D	2.077(12)	P1-O1	1.516(9)
Zr1-O6E	2.077(12)	P1-O2	1.513(9)
Zr2-O4D	1.827(11)	P2-O4	1.361(10)
Zr2-F1	1.926(11)	P2-O6	1.506(10)
Zr2-F6	1.927(10)	P2-O5	1.632(12)
Zr2-F2	1.999(12)	P3-O9	1.430(10)
Zr2-O2	2.026(8)	P3-O8	1.467(11)
Zr2-O8F	2.317(13)	P3-O7	1.659(13)
Zr3-F5	1.990(9)	P4-O12	1.451(10)
Zr3-F4	1.996(9)	P4-O11	1.471(12)
Zr3-F3	2.029(9)	P4-O10	1.519(11)

Symmetry transformations used to generate equivalent atoms for **SZ-1**: A: -x+1, -y+1, -z+1; B: -x+1/2, y+1/2, z; C: x+1/2, -y+1/2, -z+1; D: x, y+1, z; E: -x+1, -y, -z+1; F: x, -y+1/2, z-1/2; G: x+1/2, y, -z+3/2; H: x, -y+1/2, z+1/2.

Supplementary Table 3. Selected bond distances (Å) and angles (o) in **SZ-2.**

Zr1-F2	1.955(4)	P1-O3	1.517(5)
Zr1-F3	1.966(4)	P1-O2	1.511(5)
Zr1-F1	1.977(4)	P1-O1	1.495(5)
Zr1-O7A	2.090(4)	P1-C4	1.792(5)
Zr1-O1B	2.106(5)	P2-O4	1.515(5)
Zr1-O5	2.094(3)	P2-O5E	1.513(3)
Zr2-F4	1.963(5)	P2-O5	1.513(3)
Zr2-F5	1.995(5)	P2-C7	1.801(6)
Zr2-O6C	2.076(5)	P3-O7	1.509(4)
Zr2-O2B	2.076(4)	P3-O7E	1.509(4)
Zr2-O2D	2.076(4)	P3-O6	1.531(6)
Zr2-O4	2.068(4)	P3-C11	1.801(6)

Symmetry transformations used to generate equivalent atoms for **SZ-2**: A: -y, x, -z; B: y-1/2, -x+1/2, -z+1/2; C: -y, x, z; D: y-1/2, -x+1/2, z-1/2; E: x, y, -z.

Supplementary Table 4. Selected bond distances (Å) and angles (o) in **SZ-3.**

Zr1-F3	1.885(16)	Zr4-O10	2.035(7)
Zr1-F2	1.926(15)	Zr4-O10I	2.035(7)
Zr1-F1	1.928(15)	Zr4-O6J	2.067(6)
Zr1-O8A	2.112(10)	Zr4-O6K	2.067(6)
Zr1-O1	2.152(12)	P1-O3	1.468(10)
Zr1-O12B	2.171(11)	P1-O2	1.486(8)
Zr2-F4	1.930(10)	P1-O1	1.510(13)
Zr2-F6	1.956(9)	P1-C1	1.805(10)
Zr2-F5	1.988(9)	P2-O4	1.497(9)
Zr2-O4	2.051(8)	P2-O5	1.502(9)
Zr2-O9C	2.096(8)	P2-O6	1.540(7)
Zr2-O3D	2.104(9)	P2-C7	1.775(9)
Zr3-F8	1.936(10)	P3-O7	1.502(10)
Zr3-F7	1.956(9)	P3-O9	1.505(10)
Zr3-F9	1.996(9)	P3-O8	1.512(10)
Zr3-O5E	2.047(8)	P3-C13	1.812(10)
Zr3-O7	2.099(8)	P4-O11	1.463(10)
Zr3-O11F	2.104(9)	P4-O10	1.486(8)
Zr4-O2G	2.028(8)	P4-O12	1.492(12)
Zr4-O2H	2.028(8)	P4-C19	1.801(10)

Symmetry transformations used to generate equivalent atoms for **SZ-3**: A: x+1, y, z; B: x+1, y, z+1; C: 3 -x+1/2, y-1/2, -z+3/2; D: -x+3/2, y-1/2, -z+3/2; E: -x+1/2, y+1/2, -z+3/2; F: x, y, z+1; G: -x+1, -y+1, -z+1; H: x-1, y, z-1; I: -x, -y+1, -z; J: -x+1/2, y+1/2, -z+1/2; K: x-1/2, -y+1/2, z-1/2.

Supplementary Table 5. EDS analysis results of **SZ-1** and **SZ-2**.

Element	*Wt% (SZ-1)*	*At% (SZ-1)*	*Element*	*Wt% (SZ-2)*	*At% (SZ-2)*
CK	50.85	69.92	***CK***	54.02	73.58
NK	04.42	05.21	***NK***	05.32	06.21
OK	12.04	12.43	***OK***	09.71	09.92
FK	06.34	05.51	***FK***	04.97	04.28
BrL	00.82	00.17	***BrL***	03.64	00.75
PK	06.63	03.54	***PK***	04.49	02.37
ZrL	16.81	03.04	***ZrL***	14.59	02.62
AuM	02.09	00.18	***AuM***	03.26	00.27
ClK	00.00	00.00	***ClK***	00.00	00.00
Matrix	Correction	ZAF	***Matrix***	Correction	ZAF

Supplementary Table 6. Weight of **SZ-1** before and after soaked in fuming acids.

	W%	Concen. (mol/L)	Before (mg)	After (mg)	Recovery (%)
aqua regia	-		100.4	95.7	95.3
oleum	95	17.8	101.2	97.9	96.7
HNO_3	66.5	14.9	102.1	99.2	97.2
HCl	37.5	12.2	103.2	97.7	94.7

Supplementary Table 7. Sorption of uranium in presence of large excess of coexisting metal cations using **SZ-2**.

Metal ions	Ratio (M/U)	U removal %
Li^{+}	11.74	100
Mg^{2+}	11.16	100
Cu^{2+}	8.48	99.67
Pb^{2+}	11.5	98.25
Al^{3+}	11.68	96.14
Ho^{3+}	14.12	82.78
Mo^{6+}	8.367	98.62

Ratio(M/U): molar ratio of metal to uranyl ions.

Supplementary Table 8. EDS analysis results of **SZ-2** and **SZ-2-U**.

Element	*Wt% (SZ-2)*	*At% (SZ-2)*	*Element*	*Wt% (SZ-2-U)*	*At% (SZ-2-U)*
CK	52.38	69.32	***CK***	37.16	58.69
NK	08.59	09.75	***NK***	07.43	10.06
OK	11.64	11.56	***OK***	17.58	20.85
FK	04.51	03.78	***FK***	01.87	01.87
PK	05.00	02.56	***PK***	05.97	03.65
ZrL	17.12	02.98	***ZrL***	19.40	04.03
UM	00.76	00.05	***UM***	10.59	00.84
Matrix	Correction	ZAF	***Matrix***	Correction	ZAF

Supplementary Table 9. Bond parameters of U-O obtained from EXAFS experimental data.

**Sample**	**Bond type**	**Coordination Number**	**Bond length R(Å)**	**Bond disorder $\sigma^2\times10^{-3}$(Å^2)**	**R factor**
Hydrated uranium	$U\text{-}O_{ax}$	2	1.77±0.02	1.2±0.4	0.005
	$U\text{-}O_{eq}$	5.0±0.4	2.42±0.02	5.4±0.8	
**SZ-2-U**	$U\text{-}O_{ax}$	2	1.78±0.02	1.1±0.5	0.008
	$U\text{-}O_{eq}$	4.4±0.5	2.39±0.02	9.0±1.0	

Supplementary Table 10. The fitting parameters of XPS data for **SZ-2-U**

Fitting parameters	U^{VI}
BE of $U4f_{7/2}$; [FWHM] (eV)	381.81 [1.96]
BE of $U4f_{5/2}$; [FWHM] (eV)	392.71 [1.92]
4f peak separation (eV)	10.90
Satellite of $U4f_{7/2}$; [FWHM] (eV)	385.05 [2.21]
Satellite of $U4f_{5/2}$; [FWHM] (eV)	395.71 [1.93]; 401.31 [3.50]
Satellite of $U4f_{7/2}$ from main peak (eV)	3.24
Satellite of $U4f_{5/2}$ from main peak (eV)	3.00
Area of $U4f_{7/2}$	1059.44
Area of $U4f_{5/2}$	817.15
Ratio of $U4f_{5/2}$:$U4f_{7/2}$	0.771

Supplementary References

1. He Y, Xiang S, Chen B. A Microporous Hydrogen-Bonded Organic Framework for Highly Selective C_2H_2/C_2H_4 Separation at Ambient Temperature. *J Am Chem Soc* **133**, 14570-14573 (2011).

2. Zaręba JK, Białek MJ, Janczak J, Zoń J, Dobosz A. Extending the Family of Tetrahedral Tectons: Phenyl Embraces in Supramolecular Polymers of Tetraphenylmethane-based Tetraphosphonic Acid Templated by Organic Bases. *Cryst Growth Des* **14**, 6143-6153 (2014).

3. Jones KME, Mahmoudkhani AH, Chandler BD, Shimizu GKH. An adamantane-based tetraphosphonic acid that forms an open diamondoid net via a hydrogen-bonded phosphonic acid-water cluster. *CrystEngComm* **8**, 303-305 (2006).

4. Taylor JM, Mahmoudkhani AH, Shimizu GKH, Taylor JM, Mahmoudkhani AH. A tetrahedral organophosphonate as a linker for a microporous copper framework. *Angew Chem Int Ed* **46**, 809–812 (2007).
